# Seascape Genomics of the Smooth Hammerhead Shark *Sphyrna zygaena* Reveals Regional Adaptive Clinal Variation

**DOI:** 10.1002/ece3.70644

**Published:** 2024-12-12

**Authors:** D. L. Grobler, J. D. Klein, M. L. Dicken, K. Mmonwa, M. Soekoe, M. van Staden, S. B. Hagen, S. N. Maduna, A. E. Bester‐van der Merwe

**Affiliations:** ^1^ Molecular Breeding and Biodiversity Group, Department of Genetics Stellenbosch University Stellenbosch South Africa; ^2^ KwaZulu‐Natal Sharks Board Umhlanga Rocks KwaZulu‐Natal South Africa; ^3^ Institute for Coastal and Marine Research (CMR), ocean Sciences Campus Nelson Mandela University Gqeberha South Africa; ^4^ The World Wild Fund for Nature (WWF) South Africa, Newlands Office Newlands, Cape Town South Africa; ^5^ Division of Marine Research Reel Science Coalition Cape Town South Africa; ^6^ Department of Ecosystems in the Barents Region, Svanhovd Research Station Norwegian Institute of Bioeconomy Research – NIBIO Svanvik Norway

**Keywords:** conservation management, elasmobranchs, genetic structure, genomics, genotype‐environment association

## Abstract

Globally, hammerhead sharks have experienced severe declines owing to continued overexploitation and anthropogenic change. The smooth hammerhead shark *Sphyrna zygaena* remains understudied compared to other members of the family Sphyrnidae. Despite its vulnerable status, a comprehensive understanding of its genetic landscape remains lacking in many regions worldwide. The present study aimed to conduct a fine‐scale genomic assessment of *Sphyrna zygaena* within the highly dynamic marine environment of South Africa's coastline, using thousands of single nucleotide polymorphisms (SNPs) derived from restriction site‐associated DNA sequencing (3RAD). A combination of differentiation‐based outlier detection methods and genotype‐environment association (GEA) analysis was employed in *Sphyrna zygaena* . Subsequent assessments of putatively adaptive loci revealed a distinctive south to east genetic cline. Among these, notable correlations between adaptive variation and sea‐surface dissolved oxygen and salinity were evident. Conversely, analysis of 111,243 neutral SNP markers revealed a lack of regional population differentiation, a finding that remained consistent across various analytical approaches. These results provide evidence for the presence of differential selection pressures within a limited spatial range, despite high gene flow implied by the selectively neutral dataset. This study offers notable insights regarding the potential impacts of genomic variation in response to fluctuating environmental conditions in the circumglobally distributed *Sphyrna zygaena* .

## Graphical abstract

The present study conducted a fine‐scale genomic assessment of the globally threatened smooth hammerhead shark *Sphyrna zygaena* , within the regional context of South Africa. The analysis of 3RAD‐seq derived SNPs revealed a distinctive genetic cline from south to east, with adaptive variation showing correlations with environmental factors such as sea‐surface dissolved oxygen and salinity. Interestingly, while neutral SNP markers indicated high gene flow and lack of regional population differentiation, the presence of differential selection pressures suggests the potential impact of genomic variation in response to changing environmental conditions in *S. zygaena* .

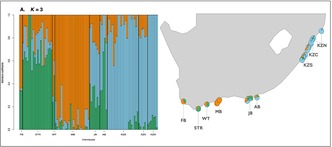

## 1 Introduction

The interaction between natural selection and gene flow has been a central focus in evolutionary biology, captivating researchers for decades. A substantial body of theoretical and empirical studies has aimed to elucidate how these forces shape genetic diversity and govern processes such as local adaptation and speciation (Haldane 1948; Slatkin 1973; Lenormand 2002; Kawecki and Ebert 2004; Savolainen, Lascoux, and Merilä 2013). Focus has been placed on investigating populations within varying demographic scenarios, spanning ecologically heterogeneous environments, including diverse seascapes, with the premise that variable selection pressures could facilitate adaptive divergence (Hendry, Taylor, and McPhail 2002; Hendry and Taylor 2004; Conover et al. 2006; Sanford and Kelly 2011; Wadgymar et al. 2022). While it is widely accepted that limited gene flow in relation to diversifying selection strength can facilitate adaptive divergence (Lenormand 2002; Garant, Forde, and Hendry 2006), classical models similarly predict that high dispersal and subsequent gene flow can lead to the swamping of locally adaptive alleles (Bulmer 1972). However, empirical assessments continuously illustrate the complexity of the interaction between gene flow and adaptive divergence (Garant, Forde, and Hendry 2006). High levels of gene flow have been shown to promote local adaptation by spreading advantageous alleles and act as a source of new genetic variation (Tigano and Friesen 2016), as in the case of genetic rescue initiatives (Hufbauer et al. 2015; Whiteley et al. 2015). Additionally, signatures of local adaptation have been detected across relatively small spatial scales (Richardson et al. 2014) or persisted within highly genetically connected populations (Clarke et al. 2010; Fitzpatrick et al. 2014; Wilder et al. 2020), implicating the importance of standing genetic variation and underlying genomic architecture in the adaptation response (Barrett and Schluter 2008; Jones et al. 2012; Tigano and Friesen 2016; Shi et al. 2021; Rougemont et al. 2022).

Traditionally, molecular assessments focused on gene flow and neutral population structure (Allendorf, Hohenlohe, and Luikart 2010; Dudgeon et al. 2012), with analyses often constrained by the availability and statistical power of only a handful of molecular markers, such as tens of nuclear microsatellite markers (Ljungqvist, Åkesson, and Hansson 2010; Putman and Carbone 2014), or a limited number of sampled individuals (Narum et al. 2014; Hohenlohe, Funk, and Rajora 2020). However, high throughput sequencing approaches such as restriction‐site associated DNA sequencing (RAD‐seq) have since enabled assessments to be conducted at a much higher genomic resolution (Narum et al. 2014; Andrews et al. 2016; Catchen et al. 2017), offering a comprehensive view of genome‐wide variation and population genetic structure across diverse taxa and wide natural biogeographical contexts, including several elasmobranchs species (Díaz‐Jaimes et al. 2021; Nikolic et al. 2022; Lesturgie et al. 2023). Furthermore, the integration of these genomic approaches with advances in remote sensing and increasing resolution of oceanographic data has led to the marked rise in studies adopting a seascape genomics framework (Grummer et al. 2019; Daupin et al. 2023). Specifically, by correlating genomic data with environmental factors (e.g., genotype‐environment association analysis), seascape genomics aims to leverage strong environmental gradients, such as those along coastlines, to identify specific drivers of neutral and adaptive genomic variation within and among marine populations (Riginos et al. 2016; Liggins, Treml, and Riginos 2020). In addition, it could provide valuable insights regarding the underlying molecular mechanisms facilitating the adaptation response (Selmoni et al. 2021; Wang et al. 2024). For instance, Skovrind et al. (2023) recently demonstrated the utility of a seascape genomics approach by identifying genomic regions that are putatively linked to genes associated with salinity tolerance. These findings suggest that such genetic adaptations likely facilitated the colonization of perch to the fluctuating salinity levels of the Baltic Sea (Skovrind et al. 2023). However, despite the demonstrated success of seascape genomics in various other marine taxa (Selmoni et al. 2021; Dorant et al. 2022; Mendoza‐Portillo, García‐de León, and von der Heyden 2023; Lowell et al. 2023), such approaches remain underutilized in highly mobile apex predators such as elasmobranchs, even though environmental fluctuations have been documented to affect their abundance and distribution patterns (Schlaff, Heupel, and Simpfendorfer 2014; Yates et al. 2015; Lee et al. 2018). In this case, the challenge lies in the difficulty of capturing the extensive spatial and temporal scales over which many marine species interact with dynamic environmental gradients (Grummer et al. 2019; Liggins, Treml, and Riginos 2020). Despite these challenges, Klein et al. (2024) recently demonstrated how fluctuations in sea‐surface salinity and other ecologically divergent selection pressures could contribute to patterns of adaptive divergence in the copper shark *Carcharhinus brachyurus* in southern Africa. Such research further underscores the potential of seascape genomics to reveal complex patterns of adaptive variation, even in shark species with wide‐ranging distributions (Klein et al. 2024). Integrating seascape genomics into conservation strategies is crucial, as this would not only facilitate the preservation of unique genetic entities, but also safeguard their adaptive potential (Funk et al. 2012; Flanagan et al. 2017; Xuereb et al. 2020). This is particularly vital for marine biodiversity, as such strategies could aid vulnerable populations—including many elasmobranch species—adapt to future environmental changes and anthropogenic threats (Liggins, Treml, and Riginos 2020; Xuereb et al. 2020; Van Oppen and Coleman 2022).

In the highly diverse marine environment of southern Africa (Tittensor et al. 2010; Lucifora, García, and Worm 2011; Worm and Branch 2012), the Agulhas and Benguela ocean current systems significantly influence distribution patterns and levels of population differentiation in various non‐model marine species (Henriques et al. 2014, 2015, 2016; Nielsen et al. 2020), including sharks (Bitalo et al. 2015; Maduna et al. 2016; Bester‐van Der Merwe et al. 2017; Kuguru et al. 2019). Fluctuations in regional abiotic conditions and biotic sea‐surface concentrations (Lutjeharms et al. 1996; Lutjeharms 2006), coupled with the projected influence of climate change, including increased sea‐surface temperatures (Hoegh‐Guldberg et al. 2007; Rouault, Pohl, and Penven 2010; Potts, Götz, and James 2015; Popova et al. 2016; Jury 2020) and ocean acidification (Orr et al. 2005; Hoegh‐Guldberg et al. 2007; Popova et al. 2016), emphasize the need to investigate the impact of such environmental shifts on the marine environment (Ramírez et al. 2017; Bas et al. 2024). This understanding is crucial for especially vulnerable populations (Osgood, White, and Baum 2021; Rodriguez‐Burgos et al. 2022) such as hammerhead sharks (family Sphyrnidae) and the overall functioning of the marine ecosystem (Hoegh‐Guldberg and Bruno 2010; Poloczanska et al. 2013; Bryndum‐Buchholz et al. 2018).

Given their high dispersal capability, highly mobile elasmobranch species, including large‐bodied hammerhead sharks, are often expected to display genetic homogeneity (Palumbi 1994; Santos and Coelho 2018). Indeed, some evidence of regional panmixia has been observed in several circumglobally distributed species, revealing only minimal genetic differentiation across ocean basins (Rus Hoelzel et al. 2006; Vignaud et al. 2014; Taguchi et al. 2015; Veríssimo et al. 2017; Junge et al. 2019). Conversely, certain elasmobranch species exhibit a wide range of behaviors influenced by habitat preference (e.g., residency, site fidelity, and philopatry) (Chapman et al. 2015), as well as environmental factors and biogeographic features (Schlaff, Heupel, and Simpfendorfer 2014; Yates et al. 2015; Lee et al. 2018), often contributing to unexpected patterns of genetic structure (Hirschfeld et al. 2021), and evidence of local adaptation (Momigliano et al. 2017), most notably within species of Sphyrnidae (Portnoy et al. 2015; Díaz‐Jaimes et al. 2021; Félix‐López, Rocha‐Oliverares, and Saavedra‐Sotelo 2024).

Hammerhead sharks occupy higher trophic levels as meso‐ or apex predators and are wide‐ranging warm‐temperate and tropical species found in continental and insular regions. Within the family Sphyrnidae, significant regional declines have been observed, especially among the large‐bodied *Sphyrna zygaena* , the great hammerhead shark *S. mokarran* , and the scalloped hammerhead shark *S. lewini* (Baum et al. 2003; Ferretti et al. 2008; Roff et al. 2018; Pacoureau et al. 2021), found in southern Africa (Ebert, Wintner, and Kyne 2021). These species are frequently targeted and caught as bycatch in pelagic longline and commercial line fisheries (Zeeberg, Corten, and de Graaf 2006; da Silva et al. 2015; Okes and Sant 2019; Thomas et al. 2021), supplying a significant component of the fin trade (Dent and Clarke 2015; Fields et al. 2018; Cardeñosa et al. 2022). In addition to these significant fishing pressures, the mortality rates associated with high capture stress further threaten the survival of these species (Gallagher et al. 2014; Ellis, McCully Phillips, and Poisson 2017). Compounding these issues, these species are often broadly categorized as multiple hammerhead shark species, “Hammerhead sharks nei,” or “Unspecified Hammerhead” in catch data (Dicken et al. 2018; Okes and Sant 2019), posing further challenges for conducting species‐specific stock assessments (Clarke et al. 2006) and accurately monitoring hammerhead shark population trends.

For *S. zygaena* , previous investigations suggest the presence of mito‐nuclear discordance, with plausible evidence for female philopatry and male‐mediated dispersal (Testerman 2014; Bolaño‐Martínez et al. 2019; Félix‐López et al. 2019), similar to observations made for other hammerhead shark species (Portnoy et al. 2015; Rangel‐Morales et al. 2022). However, barring a recent population genomic investigation (Félix‐López, Rocha‐Oliverares, and Saavedra‐Sotelo 2024), molecular assessments of the circumglobally distributed *S. zygaena* [Compagno 1984] remain limited relative to other large‐bodied Sphyrnidae. For these other species, recent genomic analyses suggest high levels of genetic connectivity at regional scales (Green et al. 2022; Brunjes et al. 2024), although some fine‐scale structuring has been detected in the Eastern Pacific (Elizondo‐Sancho et al. 2022). While the only previous population genetics study in the region detected some genetic discontinuity among south (Mossel Bay) and east (Algoa Bay and KwaZulu‐Natal) *S. zygaena* populations, it is important to note that this study also relied on a limited set of microsatellite loci (*n* = 7) and sampling locations (*N* = 3) (Kuguru et al. 2019). To address these limitations and build upon previous research, the present study aimed to conduct a fine‐scale genomic assessment of the circumglobally distributed apex predator, the smooth hammerhead shark *S. zygaena* , within an extended regional context of South Africa. Thousands of SNPs generated through restriction‐site associated DNA sequencing (3RAD‐seq) were used to characterize patterns of neutral and adaptive genetic variation among *S. zygaena* individuals sampled along the highly heterogeneous south and east coast of South Africa. Furthermore, the influence of bioclimatic and spatial factors on putatively adaptive genetic variation was investigated to explore their potential impact on the adaptation response.

## 2 Materials and Methods

### 2.1 Sample Collection and 3RAD Sequencing

**FIGURE 1:**
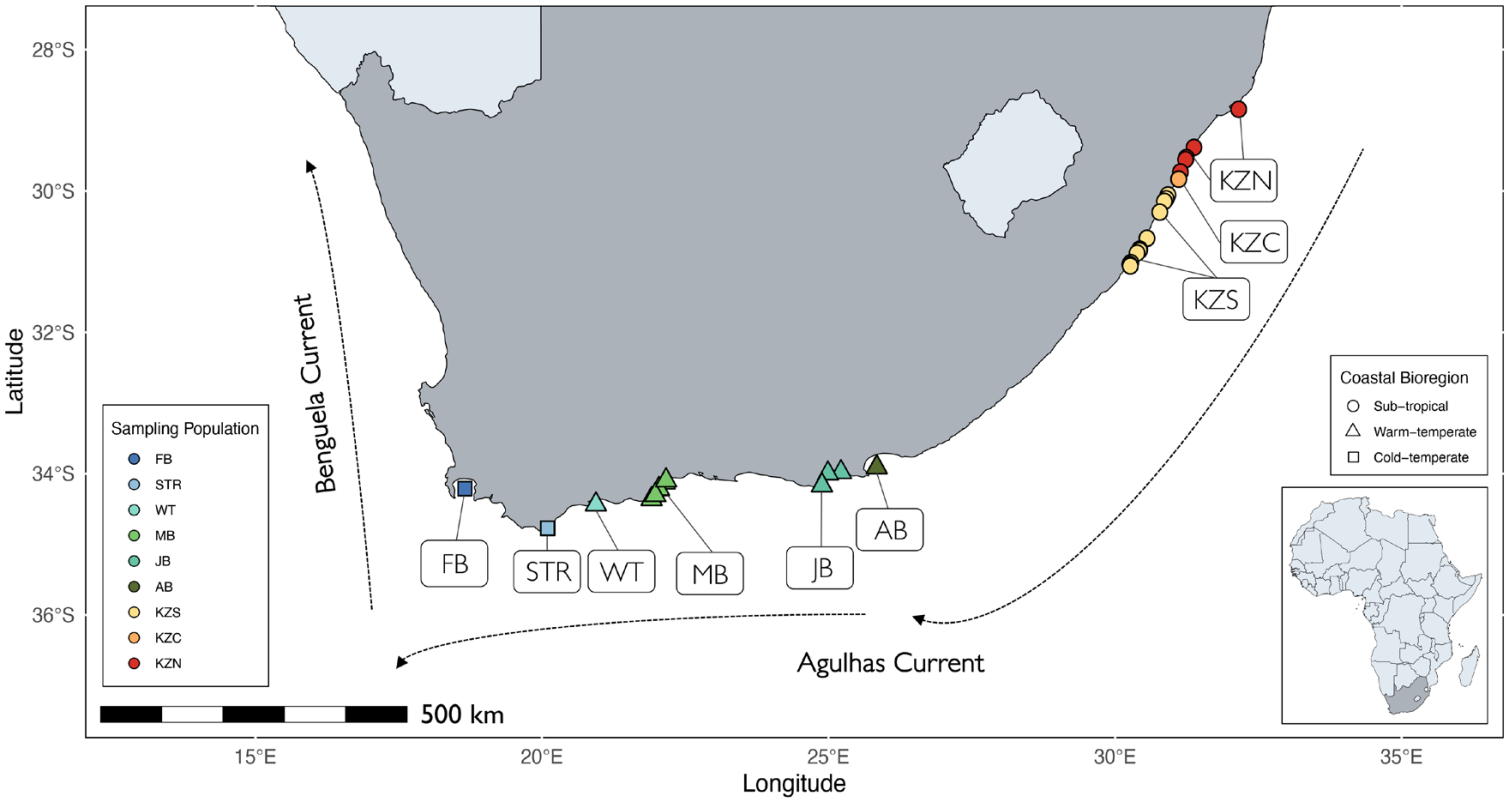
Regional sampling map of the circumglobally distributed *Sphyrna zygaena* . Fin clip samples (*n* = 95) were obtained from the south to east coast of South Africa (dark gray). The Agulhas and Benguela Currents are indicated. The main bioregions are shown, and sampling populations are color coded accordingly (adapted from Bustamante and Branch 1996; Turpie, Beckley, and Katua 2000; Potts, Götz, and James 2015). Sample labels denote: False Bay (FB), Struisbaai (STR), Witsand (WT), Mossel Bay (MB), Jeffrey's Bay (JB), Algoa Bay (AB), KwaZulu‐Natal South (KZS), KwaZulu‐Natal Central (KZC), and KwaZulu‐Natal North (KZN).

Fin clip samples were obtained from KwaZulu‐Natal Sharks Board's bather protection program and the Reel Science Coalition, comprising of recreational anglers using rod and reel methods. To note, all samples were collected from juvenile *S. zygaena* (male PCL < 210 cm; female PCL < 250 cm; Table S1). Sampling occurred opportunistically between 2008 and 2021 along the south to east coast of South Africa (Table S1) across several coastal biogeographic zones (Potts, Götz, and James 2015). All sampling was conducted in full compliance with the necessary permits and ethical approval (Research Ethics, Animal Care and Use, #ACU‐2021‐21616) (Figure 1). Upon collection, all samples were stored in 95% ethanol for further processing. The collected samples were broadly categorized into nine sampling locations according to geographic region, namely: False Bay (FB; *n* = 2), Struisbaai (STR; *n* = 20), Witsand (WT; *n* = 2), Mossel Bay (MB; *n* = 22), Jeffrey's Bay (JB; *n* = 8), Algoa Bay (AB; *n* = 5), southern KwaZulu‐Natal (KZS; *n* = 21), central KwaZulu‐Natal (KZC; *n* = 8), and northern KwaZulu‐Natal (KZN; *n* = 7). Genomic DNA was isolated using a standard cetyltrimethylammonium bromide (CTAB) method (Sambrook and Russell 2001), with the quality of DNA confirmed through gel electrophoresis (1% agarose), and the purity of samples assessed using a NanoDrop ND 2000 Spectrophotometer (Thermo Fisher Scientific, Waltham, USA). The quantification of the extracted total DNA was performed using a Qubit dsDNA Quantification Assay Kit (Thermo Fisher Scientific, Waltham, USA).

A total of 95 samples were selected for genotyping following design 2 of the 3RAD‐seq protocol (Bayona‐Vásquez et al. 2019) and a detailed description of the library preparation protocol and laboratory procedures can be found in Klein et al. (2024). Samples were pooled with a 3RAD library from another project containing 96 samples and underwent combined sequencing on a single flow cell. Sequencing was conducted using an Illumina NovaSeq 6000 platform at the Norwegian Sequencing Centre, Oslo, Norway.

### 2.2 Read Processing

All raw fastq files were extracted, and the forward and reverse libraries were merged to combine the complementary information from both ends of the sequenced fragments and improve the accuracy of downstream analyses. Reading pairs were trimmed of adapters using Cutadapt 1.91. (Martin 2011), and visually inspected using FastQC 0.11.5 (Andrews 2010) before processing in STACKS 2.59 (Catchen et al. 2011). Sequencing reads were demultiplexed and quality filtered using the *process_radtags.pl* module of STACKS. Reads containing uncalled bases were removed (*‐c*), barcodes and RAD‐Tag cut sites were rescued (*‐r*), and low‐quality reads were discarded (*‐q*). A default sliding window (*‐w*) of 15% of the read length and a raw Phred score threshold of 10 (*‐s*) were applied. Furthermore, sequencing reads were truncated to a final length of 110 bp (*‐t*).

### 2.3 De Novo Assembly

The parameters for the de novo assembly process were optimized with STACKS. The most appropriate core parameters for STACKS were selected following the approach outlined by Paris, Stevens, and Catchen (2017) and Rochette and Catchen (2017), known as the r80 method. This method focuses on maximizing the utilization of biological information, thus generating a reliable set of loci, known as r80 loci, essential for downstream analyses (Paris, Stevens, and Catchen 2017). Here, a locus is considered for processing only if it is present in a minimum of 80% of individuals within a designated population (*−r* 0.80) (Paris, Stevens, and Catchen 2017; Rochette and Catchen 2017). The *denovo_map.pl* pipeline was executed on a subset of 20 samples (Rivera‐Colón and Catchen 2022), with an adjusted phasing tolerance (*‐X: “gstacks: ‐‐phasing—cooccurrences‐thr‐range 2,3”*) to improve phasing accuracy within highly repetitive sequenced fragments, and *‐r* 0.80 was specified for each run (Paris, Stevens, and Catchen 2017; Rochette and Catchen 2017). The *ustacks M*‐parameter and *cstacks n*‐parameter were varied together from 0 to 12 (*M* = *n* = 0—*M* = *n* = 12). Additionally, *n* = *M* plus or minus one iteration was also tested as recommended by Paris, Stevens, and Catchen (2017). Once the runs were complete, the number of SNPs, polymorphic loci, and assembled loci were extracted and assessed. The optimal value of *M/n* was determined by observing the change in r80 loci, aiming for the point at which the change approaches zero while remaining positive (Paris, Stevens, and Catchen 2017). In this case, r80 loci were lost from the initial stages. Therefore, for the final dataset, the core pipeline was executed allowing a maximum of up to two mismatches within a locus at the sample level *(‐M*) and across the whole dataset (*‐n*).

### 2.4 SNP Filtering

Following de novo assembly, additional filtering was performed in the *populations* module of STACKS. Putative loci were only retained if present in at least 80% of individuals across all sampled populations (*‐R*). In addition, loci exhibiting an excess of heterozygosity (*‐‐max‐obs‐het* 0.5) or a minor allele frequency (*‐‐min‐maf*) of less than 0.01 were removed. Additional minor allele frequency thresholds were tested (0.05 and 0.1, respectively), although estimates of population differentiation did not significantly differ (*results not shown*). The amount of missing data per locus and per individual was assessed using the *‐‐missing‐site* and *‐‐missing‐indv* functions in VCFtools 0.1.17 (Danecek et al. 2011), and samples with over 40% missing data were subsequently removed. To avoid the likelihood of linkage between SNPs, only the first SNP per locus was retained. The resulting SNP dataset was exported into various formats suitable for downstream analyses using the *populations* module of STACKS and PGDSpider 2.1.1.5 (Lischer and Excoffier 2011). Finally, the *filter.sex.linked* function developed by Robledo‐Ruiz et al. (2023) was employed to test for the presence of sex‐linked loci.

### 2.5 Genetic Diversity

To evaluate genetic diversity, an analysis using the full SNP dataset with the *populations* module within STACKS was conducted. Here, private allele counts and nucleotide diversity (π) for each sampling location were assessed. Mean observed (*H*_O_) and expected heterozygosity (*H*_E_), along with fixation indices (*F*_IS_), were determined using the diveRsity 1.9.90 R‐package (Keenan et al. 2013). To note, the FB and WT sample populations were not considered due to small sample size (*n* = 2). Specifically, diveRsity was run using the *basicStats* function (Keenan et al. 2013), and 95% Confidence Intervals (CI) were calculated for each *F*_IS_ value based on 500 bootstrap permutations.

### 2.6 Identifying Neutral and Adaptive Loci

To maximize the detection of true adaptive loci, a dual approach was employed combining differentiation‐based (*F*_ST_) methods (OUTFlank 0.2 and pcadapt 4.3.5) and gene–environment association (GEA) analysis. Specifically, OUTFlank was used for its ability to minimize false positives (Whitlock and Lotterhos 2015), while pcadapt was selected for its lack of bias toward prior assumptions regarding population structure (Duforet‐Frebourg et al. 2016; Luu, Bazin, and Blum 2017). Additionally, multivariate redundancy analysis (RDA) was incorporated to detect loci correlated with environmental gradients, aiming to leverage the strengths of each method to provide a more comprehensive assessment of selection signals across the genome (Forester et al. 2018).

For the GEA analysis, the multivariate Redundancy Analysis (RDA) method was employed (Forester et al. 2018; Capblancq and Forester 2021). The individual‐based datasets (genotype matrix) were treated as response variables, and any missing values were imputed by using the most common allele at each locus across all sampled individuals (Forester et al. 2018). To account for potential confounding effects of spatial structure within the dataset (Lotterhos and Whitlock 2015), explanatory factors including longitude and latitude, in addition to climate variables were considered.

Geographic distance matrices were created by calculating the least‐cost distances via seas (avoiding landmasses) between sampling sites using the *lc.dist* function from the R package marmap 1.0.10 (Pante and Simon‐Bouhet 2013). The resulting matrices were converted to Euclidean distances and used to compute distance‐based Moran's eigenvector maps (dbMEMs) using the adespatial 0.3 package (Dray et al. 2023). In terms of climate variables, a total of 21 ecologically relevant environmental factors (Schlaff, Heupel, and Simpfendorfer 2014; Yates et al. 2015; Lee et al. 2018), including salinity (e.g., MS_biogeo08_sss_mean_5m), dissolved oxygen (e.g., BO22_dissoxmax_ss), temperature (e.g., MS_biogeo13_sst_mean_5m), and primary production (BO22_ppmean_ss), among others, were tested (Table S2). These variables were obtained from the Bio‐Oracle 2.2 (Assis et al. 2017) and Marine Spatial Ecology (MARSPEC) (Sbrocco and Barber 2013) databases via the sdmpredictors 0.2.14 package (Bosch et al. 2023). Mean, minimum, maximum, and range sea‐surface measurements were included from each sampling site when available (Table S2).

To obtain the final explanatory dataset, distance vectors and environmental variables were separately submitted to a stepwise selection procedure implemented in the *ordistep* function in vegan 2.0‐2 (Oksanen et al. 2012). Proportions of genetic variance explained solely or jointly by each set of variables were estimated by comparing the adjusted *R* ^2^ in a series of partial RDAs using distance vectors as conditioning variables. The Variance Inflation Factor (VIF) was calculated to assess multicollinearity among variables, and the significance of the model (full model and per axis) was determined through ANOVA with 1000 permutations and an alpha level of 0.05. In models showing significance, candidate loci were identified based on locus scores, which reflect the loading of each locus in ordination space. Loci were considered candidates if their scores deviated from the 97.5% Confidence Intervals (CI) of the mean loading of RDA1 and RDA2. Additionally, environmental variables with the strongest associations with each candidate adaptive locus were explored using Pearson's correlation coefficient (*r*).

OUTFlank 0.2 (Whitlock and Lotterhos 2015) and pcadapt 4.3.5 (Luu, Bazin, and Blum 2017) were used for differentiation‐based analyses. OUTFlank employs a maximum likelihood approach which calculates a likelihood based on a trimmed distribution of *F*_ST_ values to infer the distribution of *F*_ST_ for neutral markers (Whitlock and Lotterhos 2015). Considering nine spatial locations, OUTFlank was executed with default parameters using a *Q‐threshold*, *LeftTrimFraction*, and *RightTrimFraction* value of 0.05. pcadapt instead utilizes principal component analysis (PCA) to detect loci under selection, assuming markers excessively correlated with population structure are indicative of local adaptation (Luu, Bazin, and Blum 2017). The analysis was performed with an initial ordination that allowed up to 93 principal components (*K*) and was compared using a “scree plot.” The proportion of variance explained by each principal component was assessed, whereafter a list of putative adaptive loci was obtained assuming a value of *K* = 1 under an expected false discovery rate (FDR) of 0.20 using the *q* value package 2.30.0 (Storey and Tibshirani 2003).

Finally, to visualize the overlap between different outlier detection methods, the VennDiagram 1.7.3 package (Chen and Boutros 2011) was employed. Two datasets were generated: one containing selectively neutral loci (*n* = 111,243) and the other containing putatively adaptive loci (*n* = 4844). To avoid overlooking loci under weak selection, the adaptive dataset included loci identified by at least one of the three outlier detection methods (Forester et al. 2018). Population structure analyses were performed independently for both datasets. Additionally, to account for potential false positives among the identified adaptive loci, a third dataset (hereafter, *overlapping loci*) consisting of loci detected by at least two outlier detection methods (*n* = 53) was created to test the robustness of genetic variability estimates (*see Results; DAPC*).

### 2.7 Population Differentiation and Genetic Clustering

An estimate of genetic differentiation per sample population was computed utilizing Weir and Cockerham (1984) *F*_ST_, as implemented in the StAMPP 1.6.3 R‐package (Pembleton, Cogan, and Forster 2013) specifying 1000 bootstrap replications. Similarly, global *F*_ST_ was calculated for neutral and adaptive datasets using hierfstat (Goudet 2005). Resulting *p*‐values were corrected with the Benjamini–Hochberg procedure (Benjamini and Hochberg 1995) using the *p.adjust* function from stats 4.4.1. (R Core Team 2024). Mantel tests were conducted to examine potential patterns of isolation‐by‐distance (IBD) and isolation‐by‐environment (IBE). Given the detected influence of dissolved oxygen on genomic variation (*see Results*), dissolved oxygen was chosen as a proxy for environmental distance. Genetic distances were transformed to linearized *F*_ST_ values (*F*_ST_/1 − *F*_ST_), and geographic and environmental distance matrices (between sampled population pairs) were calculated using the *lc.dist* function (Pante and Simon‐Bouhet 2013) as described above, and the *veg.dist* function (Oksanen et al. 2012), respectively. The analysis was conducted using the vegan 2.0‐2 R‐package (Oksanen et al. 2012) with significance (*p*‐value < 0.05) assessed through 9999 permutations.

Additionally, an individual‐based genetic cluster analysis using the sparse non‐negative matrix factorization (*sNMF*) algorithm implemented in the LEA 3.10.2 package (Frichot and François 2015) was conducted. This method offers similar performance to other population software tools, while significantly reducing computational time (Frichot et al. 2014). In particular, the analysis involved testing values of *K* (number of genetic groups) ranging from 1 to 10 with five replicates and a regularization parameter (*alpha*) set to 100 for both neutral and putatively adaptive loci. The optimal *K* (*K* = 1 and 2, respectively; *see Results*) was selected based on the run with the lowest entropy value and compared to the results obtained by the model‐based, Bayesian clustering software, fastSTRUCTURE 1.0 (Raj, Stephens, and Pritchard 2014). For this, the simple prior across the same range of *K* was employed (Raj, Stephens, and Pritchard 2014). Additionally, the *chooseK.py* function was used to determine the probable range of *K* by identifying the model components that cumulatively contribute to at least 99% of ancestry and maximize the log‐marginal likelihood lower bound (LLBO) of the data.

Finally, a discriminant analysis of principal components (DAPC) using the R package adegenet 2.1.10 (Jombart and Ahmed 2011) was performed, which offers the advantage of identifying potential genetic clines and hierarchical structures without making presumptions about the underlying population genetic models (Jombart, Devillard, and Balloux 2010). The DAPC analysis was conducted both with and without specifying a priori population designations. Specifically, given that the model selection indicated *K* = 1 as the most likely number of clusters (*see Results*), DAPC was also run with collection localities as prior groupings to visualize genetic variability. For the former, cluster identification was performed using the *find. clusters* function, with the optimal number of genetic groups evaluated using the Bayesian Information Criterion (BIC). To avoid overfitting, cross‐validation (*xvalDAPC*) was performed using 90% of the data as a training set with 100 replicates and selected the number of retained PCs based on the lowest mean square error (MSE) obtained.

## 3 Results

### 3.1 Read Processing and Filtering

The 3RAD sequencing procedure produced a total of 2,500,491,098 raw paired‐end reads. After quality control, the STACKS catalog was built from the remaining 1,685,147,915 reads (67.393%), with an average depth of 28.440 across all samples. Subsequent filtering using the *populations* module in STACKS and VCFtools resulted in 111,243 autosomal SNPs across 93 samples and 9 sampling locations.

### 3.2 Genetic Diversity

Patterns of genetic diversity were largely congruent among all sampling locations despite the large discrepancy in sample size (5–23), which was reflected in the number of private alleles (53–2313) (Table 1). Nonetheless, nucleotide diversity remained low across all populations (*π* = 0.115–0.135), with AB recording the highest (*π* = 0.135) (Table 1). All populations exhibited negative values of *F*_IS_ significantly different from zero, with highest values observed for STR and MB populations (*F*_IS_ = −0.001 and − 0.003, respectively).

**TABLE 1:** Summary of statistics of the full SNP dataset for all sampled populations of *Sphyrna zygaena* . To note, FB and WT sample populations (*n* = 2) were not considered for population‐level diversity statistics. The private allele count, and nucleotide diversity (*π*) were determined using the *populations* module of STACKS. Mean observed (*H*_O_) and expected heterozygosity (*H*_E_) in addition to the inbreeding coefficients (*F*_IS_) and corresponding 95% Confidence Intervals (CI) are shown.

Location	Sampled individuals	Private alleles	Nucleotide diversity (π)	*H*_O_	*H*_E_	*F*_IS_ (95%CI)
STR	20	1738	0.118	0.115	0.115	−0.003 (−0.040; −0.009)
MB	23	2313	0.117	0.113	0.114	−0.001 (−0.029; −0.009)
JB	7	281	0.119	0.122	0.109	−0.105 (−0.286; −0.110)
AB	5	53	0.135	0.159	0.133	−0.315 (−0.648; −0.315)
KZS	21	1870	0.118	0.123	0.107	−0.124 (−0.096; −0.051)
KZC	8	271	0.115	0.112	0.105	−0.076 (−0.312; −0.086)
KZN	7	211	0.117	0.123	0.107	−0.124 (−0.331; −0.130)

### 3.3 Identification of Loci Putatively Under Selection

**FIGURE 2:**
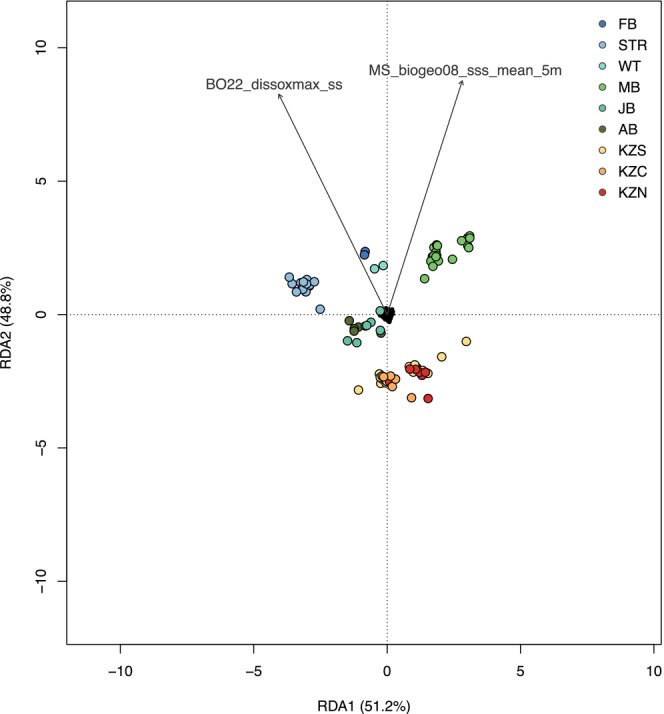
Partial Redundancy analysis (RDA) testing for the association between retained environmental and spatial variables, and genomic variation in *Sphyrna zygaena* individuals (*n* = 93) along the south to east coastline of South Africa. Dots correspond to individuals, which are colored by sampling location. Analyses were performed using the selected environmental variables (indicated by gray arrows), dissolved oxygen (BO22_dissoxmax_ss) and salinity (MS_biogeo08_sss_mean_5m) sea‐surface concentrations, with four spatial variables (MEM38, MEM47, MEM50 and MEM51) included as conditioning variables.

For RDA, the *ordistep* selection procedure included the sea‐surface environmental variables, dissolved oxygen (BO22_dissoxmax_ss) and salinity (MS_biogeo08_sss_mean_5m) (Figure S4), while the four selected distance vectors, MEM38, MEM47, MEM50, and MEM51 were retained as conditioning variables. One variable (MEM1) was excluded due to high collinearity (VIF ~ 10) (Table S3). The final partial RDA model was significant on both the full model (ANOVA, *p*‐value = 0.047) and per axis levels (ANOVA, *p‐*value = 0.032 and 0.049, respectively). Analyzing the distribution of samples within the ordination space revealed distinct patterns: samples from the MB region displayed a positive association with salinity levels compared to those in KZN (Figure 2). Furthermore, FB, STR, and WT samples displayed a positive correlation with dissolved oxygen levels, while samples from KwaZulu–Natal (KZS, KZC, and KZN) displayed a negative correlation with levels of dissolved oxygen at the sea surface (Figure 2). Collectively, dissolved oxygen and salinity contributed to 2.26% of the explained variance (adjusted *R* ^2^ = 0.099%), whereas the distance conditioning variables accounted for 4.70%. The final partial RDA model identified a total of 3865 putative adaptive loci, where 2183 loci were most strongly correlated to dissolved oxygen and 1652 to salinity sea‐surface concentrations.

Considering *F*_ST_ outlier detection methods, a total of 1033 SNPs were identified as putatively adaptive. OUTFlank was the most conservative, detecting 368 SNPs, with pcadapt identifying 665.

Overall, a total of 4844 unique loci (4.173%) were identified by at least one outlier detection method (Figure S1). A single locus was identified by all three approaches, while 53 were identified by at least two (Figure S1).

### 3.4 Population Differentiation

**FIGURE 3:**
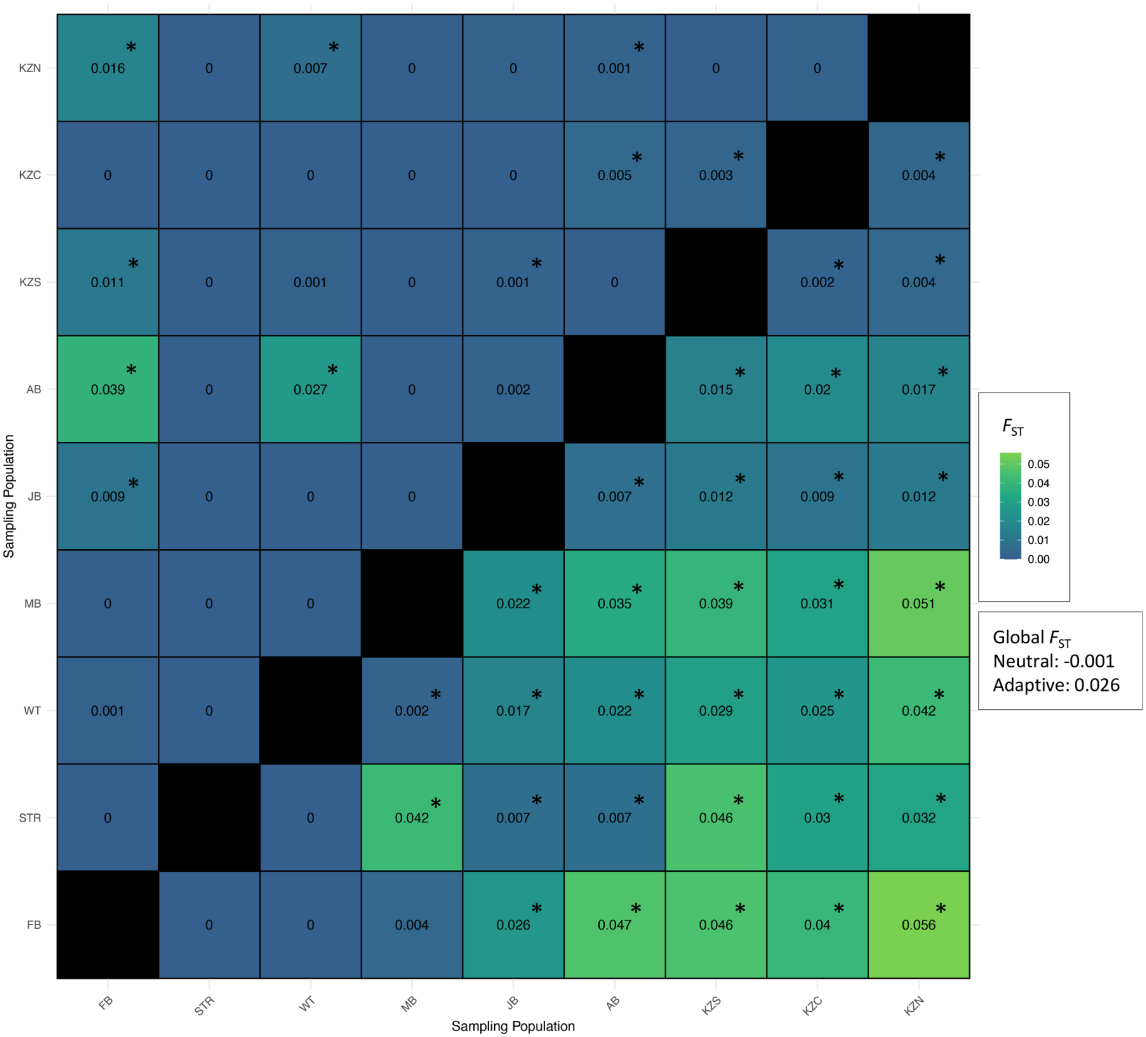
Heatmap representing pairwise *F*_ST_ comparisons between all *Sphyrna zygaena* sampled populations (*n* = 9). Global *F*_ST_ values are indicated. The diagonal is represented by gray tiles. Values above the diagonal correspond to neutral loci (*N* = 111,243), while the lower diagonal depict putatively adaptive loci (*N* = 4844). Significance (*p*‐value < 0.05) is represented by an asterisk.

Based on selectively neutral loci, pairwise measures of genetic differentiation (*F*_ST_) between sampling populations were low, yet statistically significant (*p*‐value < 0.05; Figure 3, upper diagonal). For the putatively adaptive loci, higher differentiation was generally observed as opposed to the comparisons of the selectively neutral dataset (Figure 3, lower diagonal), in congruence with global *F*_ST_ (*F*_ST_ = 0.0261 and −0.001, respectively). Notable among these comparisons was the moderate differentiation between MB and KZN sample populations (*F*_ST_ = 0.051; *p*‐value < 0.05; Figure 3). Additionally, patterns emerged where populations sampled in closer proximity, such as KZS and KZC (*F*_ST_ = 0.002; *p*‐value < 0.05), displayed lower *F*_ST_ values (Figure 3). Conversely, populations separated by greater geographic distances, such as STR and KZS (*F*_ST_ = 0.046; *p*‐value < 0.05) displayed higher levels of differentiation (Figure 3).

**FIGURE 4:**
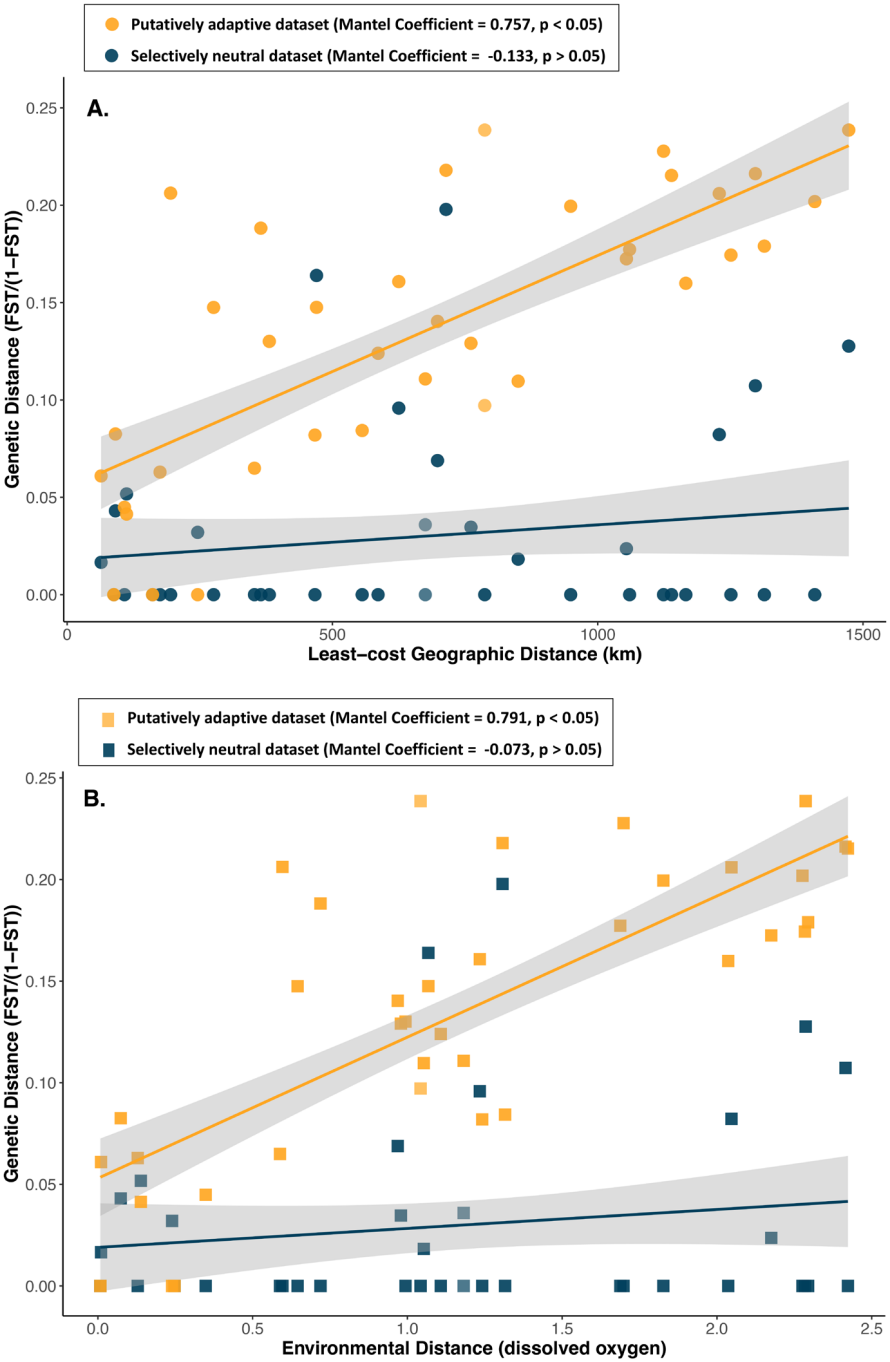
An assessment of (A) isolation‐by‐distance (IBD) and (B) isolation‐by‐environment (IBE) for both selectively neutral and putatively adaptive datasets by means of a Mantel test for *Sphyrna zygaena* . Linearized *F*_ST_ (*F*_ST_/1 − *F*_ST_) values were correlated to least‐cost geographic distance (km) and environmental distance (dissolved oxygen). Points corresponding to the neutral dataset (*n* = 111, 243) are depicted in navy, and the adaptive dataset (*n* = 4844), orange. Trend lines and 95% confidence intervals (dark gray) are shown. The Mantel Coefficient and assessment of significance (*p*‐value > 0.05) for each dataset is displayed.

The assessment of isolation‐by‐distance (IBD) revealed no statistically significant correlation (Mantel Coefficient = −0.133; *p*‐value > 0.05) when considering selectively neutral markers (*n* = 111, 243) (Figure 4A). In contrast, a significant correlation (Mantel Coefficient = 0.345; *p*‐value < 0.05) between genetic (*F*_ST_/1 − *F*_ST_) and least‐cost geographic distances (km) was evident based on the adaptive loci (*n* = 4844) (Figure 4A). A similar pattern emerged considering our assessment of isolation‐by‐environment (IBE). Similarly, our analysis of isolation‐by‐environment (IBE) revealed a high to moderate correlation between genetic and environmental distance (dissolved oxygen) in the case of the adaptive markers (Mantel Coefficient = 0.791, *p* < 0.05), a correlation which was not found to be statistically significant when considering the selectively neutral dataset (Mantel Coefficient = −0.073; *p*‐value > 0.05; Figure 4B).

Investigating the number of genetic clusters without prior knowledge of sampling localities, both LEA and fastSTRUCTURE agreed on *K* = 1 for the neutral dataset (Figure S2 and Table S4). However, in the context of putatively adaptive loci, LEA and fastSTRUCTURE suggested *K* = 3 and *K* = 2, respectively (Figure S2 and Table S4).

**FIGURE 5:**
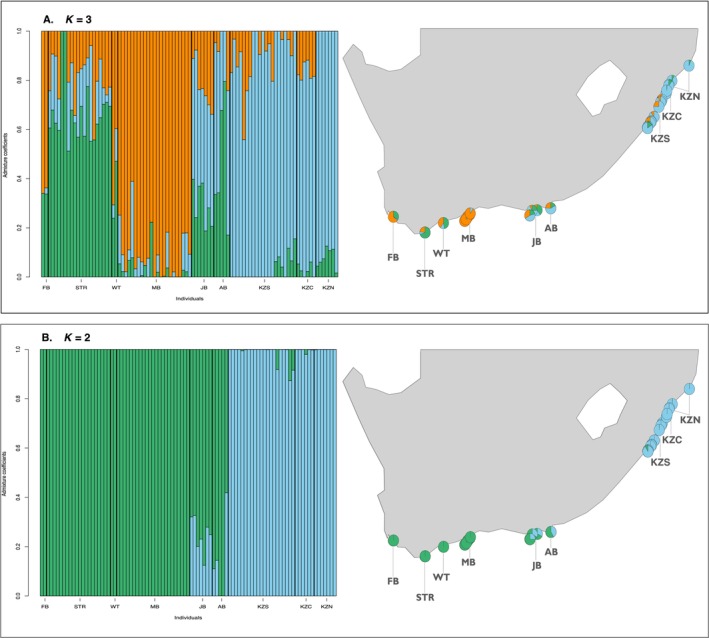
Individual admixture coefficients based on putatively adaptive loci (*n* = 4844) for *Sphyrna zygaena* (*n* = 93). Admixture estimates were determined using (A) the *sNMF* procedure in LEA and (B) likelihood estimation implemented in fastSTRUCTURE. Individuals are represented by vertical bars and ordered according to sampling location. Proportions in pie charts correspond to ancestry coefficients estimated by the respective algorithms and are plotted according to sampling location along the south to east coast of South Africa.

Both LEA and fastSTRUCTURE consistently identified genetic discontinuity among populations in the southern (FB, STR, WT, and MB) and eastern (AB, JB, KZS, KZC, and KZN) regions, although with varying degrees of observed admixture (Figure 5). LEA delineated up to three putative populations, with individuals sampled from FB and STR assigned to cluster 1, those from WT and MB assigned to cluster 2 and considerable admixture observed among individuals sampled from JB, AB, KZS, KZC, and KZN, representing a distinct third cluster (Figure 5A). In contrast, fastSTRUCTURE similarly identified genetic divergence between the southern and eastern populations, with a less pronounced level of observed admixture (Figure 5B).

The *find. clusters* analysis algorithm implemented in the DAPC was in agreement with LEA and fastSTRUCTURE, validating *K* = 1 as the optimal configuration for selectively neutral loci (Figure S3 and Table S4). The BIC criterion supported *K* = 1 also for adaptive loci (Figure S3), contrasting with the *sNMF* and fastSTRUCTURE estimates (Table S4).

**FIGURE 6:**
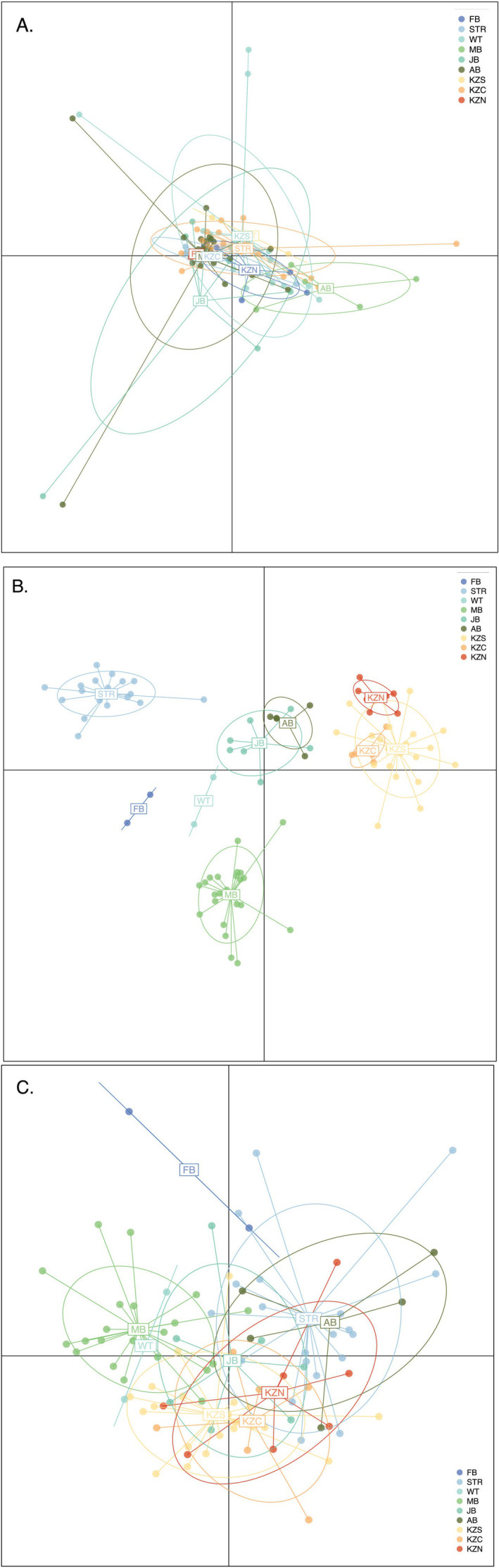
Discriminant Analysis of Principal Components (DAPC) scatterplot considering a priori population designations based on (A) 111,243 selectively neutral, (B) 4844 putatively adaptive, and (C) 53 overlapping loci. Each dot represents an individual genotype, and the 95% inertia ellipses represent each sampled population. For (A) three Principal Component axes (PCs) were retained; for (B), six PCs; and for (C) 15 PCs were retained. PCs were retained based on the lowest mean square error (MSE) determined by the cross‐validation (*xvalDAPC*) procedure in adegenet. Eigenvalues are not displayed as the DAPC analysis involved fewer retained PCs than the specified number of sampling clusters (Jombart, Devillard, and Balloux 2010).

When incorporating sampling locality as a prior, the DAPC analysis involving neutral loci (*n* = 111,243) revealed significant genetic overlap among all nine sample populations (Figure 6A). In contrast, for adaptive loci, the degree of observed genetic differentiation varied depending on the number of putatively adaptive loci considered. While some weakly differentiated clusters were observed in the overlapping adaptive dataset (*n* = 53) (Figure 6C), the less stringent adaptive dataset (*n* = 4844) exhibited more pronounced separation among sample populations (Figure 6B), likely due to the increased resolution afforded by the inclusion of additional SNP markers under weak selection. Specifically, distinct clusters were observed for False Bay (FB), Struisbaai (STR), Witsand (WT), and Mossel Bay (MB) samples (Figure 6B), while considerable overlap was consistently observed among KwaZulu‐Natal samples (KZS, KZC, and KZN), as well as between Algoa Bay (AB) and Jeffrey's Bay (JB) samples (Figure 6B,C).

## 4 Discussion

In the present study, a fine‐scale population genomic assessment of the relatively understudied smooth hammerhead shark, *S. zygaena* was conducted. Through the analysis of thousands of 3RAD‐seq‐derived SNPs, our investigation aimed to elucidate the scale and potential drivers of genetic differentiation in the globally distributed *S. zygaena* , focusing on the regional species distribution in South Africa. Notably, our analysis of neutral SNP markers showed no genetic differentiation among sampled *S. zygaena* populations. In contrast, putatively adaptive loci revealed genetic variation along a south to east spatial‐environmental cline. This pattern showed significant correlations with sea‐surface dissolved oxygen and salinity concentrations, alongside distance vectors. The present study contributes to understanding the genetic diversity of vulnerable *S. zygaena* . Furthermore, it sheds light on unique adaptations within various habitats along the diverse South African south to east coast.

### 4.1 Genetic Diversity

The heterozygosity estimates (*H*_O_ and *H*_E_) obtained in the present study were low yet remained consistent across sample populations and with findings from similar research. For example, studies employing reduced representation sequencing to investigate genetic diversity and connectivity in other shark species, including the gray reef shark *Carcharhinus amblyrhynchos* (*H*_O_: 0.139–0.301; *H*_E_: 0.133–0.302; Momigliano et al. 2017), the silvertip shark *Carcharhinus albimarginatus* (*H*_O_: 0.126–0.130; *H*_E_: 0.139–0.152; Green et al. 2019), the copper shark *C. brachyurus* (Ho: 0.170–0.195; *H*_E_: 0.149–0.167) (Klein et al. 2024) within a similar study area, and recently, another regional *S. zygaena* genomic assessment (*H*_O_: 0.080–0.513; *H*_E_: 0.099–0.497) (Félix‐López, Rocha‐Oliverares, and Saavedra‐Sotelo 2024; Table 1). Interestingly, a slight yet significant excess of heterozygosity (*F*_IS_ < 0; *p*‐value < 0.05) was observed. Theoretically, this excess could be related to the presence of overdominance or sex‐specific selection pressures (Waples 2015), with these effects potentially further amplified by the uneven sex ratios within the sampled cohort (*n* = 25 males; *n* = 52 females). Notably, previous assessments of *S. zygaena* have suggested a discrepancy between the level of genetic structure identified using matrilineally inherited mtDNA and nuclear markers. This discrepancy could potentially be attributed to female philopatry, where gene flow predominantly occurs through male‐mediated dispersal (Testerman 2014; Félix‐López et al. 2019). While this scenario could potentially introduce sex‐specific selection pressures, further investigation would be required given the lack of sex‐linked loci detected in the present dataset. Further empirical evidence, possibly through a complementary analysis of mtDNA, would be required to confirm this hypothesis (Portnoy et al. 2015).

### 4.2 Neutral vs. Adaptive Population Genomic Structure

Although recent studies have investigated the population genetic structure of several large, widely distributed shark species, including the tiger shark *Galeocerdo cuvier* (Bernard et al. 2021; Lesturgie et al. 2023), the gray reef shark *C. amblyrhynchos* (Momigliano et al. 2017), and the scalloped hammerhead shark *S. lewini* (Green et al. 2022), these examinations have primarily focused on the identification of putatively adaptive markers linked to excessive *F*_ST_‐based differentiation. However, the influence of diverse marine environments on adaptive differentiation has often not extensively been explored. Moreover, our understanding of the scale and potential drivers of genetic connectivity in many elasmobranch species, such as *S. zygaena* , remains limited.

In a similar study area, the only other regional population genetic assessment found no distinct regional differentiation based on mtDNA among *S. zygaena* sampled populations (*n* = 3) (Kuguru et al. 2019). Instead, some nuclear structure between southern (Mossel Bay) and eastern (Algoa Bay and KwaZulu‐Natal) *S. zygaena* populations was detected. In contrast, the present analysis of selectively neutral SNP markers found no regional differentiation among *S. zygaena* populations across the species distribution in South Africa. It is important to note, however, that the prior study analyzed only a limited number of microsatellite loci (*n* = 7) across three geographically sourced sample populations. In contrast, the present study benefits from increased genomic resolution afforded by thousands of RAD‐sequencing‐derived SNPs and a broader sampling regime. Therefore, the present analysis provides a more detailed estimate of genetic connectivity among regional *S. zygaena* . Nonetheless, the detected patterns of high regional connectivity based on neutral markers are consistent with those observed in the Mexican Pacific (Félix‐López, Rocha‐Oliverares, and Saavedra‐Sotelo 2024) and previous findings indicating substantial gene flow and minimal genetic differentiation among global *S. zygaena* populations (Testerman 2014). Moreover, assessments of nuclear microsatellites in another large member of the family Sphyrnidae, *S. lewini* , consistently reflect high regional genetic connectivity in the Eastern Pacific (Nance et al. 2011; Quintanilla et al. 2015; Rangel‐Morales et al. 2022), Western Atlantic (Pinhal et al. 2020), and, to some extent, the Central Indo‐Pacific (Green et al. 2022). In the case of Daly‐Engel et al. (2012), the analysis extends globally, but still found evidence supporting high genetic connectivity along coastal areas, which could further corroborate the high level of gene flow observed in the present analysis.

Alternatively, the significant temporal variation between sampling years in Mossel Bay (MB1 and MB2) observed by Kuguru et al. (2019)—combined with the fact that most Mossel Bay individuals in this study were collected in a single year (2020; Table S1)—suggests that temporal dynamics, rather than spatial structure, could account for the differences in genetic differentiation reported by the prior study and those detected in the present analysis. Additionally, given that the diversity estimates from several sample populations (MB1, MB2, and KZN) showed significant deviations from Hardy–Weinberg equilibrium (HWE) (*p*‐value < 0.05), it is possible that their estimates of genetic differentiation reflect some selective influence. In this context, their differentiation estimates may align more closely with those reported for the adaptive dataset (e.g., Figure 5B) compared to the neutral panmixia reported in the present analysis.

Interestingly, reports from a previous capture‐mark‐recapture study conducted off the eastern coast of South Africa reported limited average travel distances for *S. zygaena* (Diemer, Mann, and Hussey 2011). This is contrary to the high coastal connectivity implied by the current study and recent findings (Félix‐López, Rocha‐Oliverares, and Saavedra‐Sotelo 2024), as well as satellite‐tagging projects conducted in the Atlantic (Santos and Coelho 2018; Logan et al. 2020); however, it could be explained by the fact that recaptured individuals were mainly juveniles (male PCL < 210 cm; female PCL < 250 cm), which are known to mostly remain in coastal nursery habitats (Smale 1991; Afonso et al. 2022; Albano et al. 2023). Moreover, the travel distances reported were based on only a small subset of recaptured individuals (*n* = 20), although the maximum travel distance recorded (384 km) does show some overlap with previous estimates from tagged individuals of similar size classes (132 and 257 km) (Santos and Coelho 2018). Collectively, these findings suggest that while *S. zygaena* is capable of long‐distance migrations, they likely demonstrate periods of residency as well (Logan et al. 2020). While future studies would greatly benefit from additional multiannual tracking projects in the region, especially considering various size classes and sex‐specific data, the current assessment of selectively neutral SNP data suggests a high level of connectivity among regional *S. zygaena* populations, likely maintained by limited restrictions to dispersal leading to substantial gene flow (Figure 4).

In contrast to the low genetic differentiation observed for neutral loci, assessments involving putatively adaptive loci revealed signals indicative of adaptive differentiation (Figure 5). Specifically, both *sNMF* and fastSTRUCTURE provided evidence for adaptive differentiation, despite *find. clusters* indicating the optimal configuration as a single cluster. The inconsistency observed in detecting genetic patterns could be attributed to the limited sensitivity of *find. clusters* to discern signals of structure in cases of relatively low genetic differentiation (Miller, Cullingham, and Peery 2020). Nevertheless, despite these methodological considerations, the signals of adaptive differentiation identified by the LEA and fastSTRUCTURE analyses consistently indicated a genetic cline from south to east, with LEA‐analysis further indicating a distinct cluster for the Mossel Bay sample population.

The pattern of clinal variation observed along the coastline for the putative adaptive dataset, coupled with the absence of differentiation in selectively neutral loci, provides evidence that gene flow likely remains unrestricted. This suggests that local adaptation may be the primary driver of the observed variation, likely influenced by selection pressures imposed by a combination of spatial (distance vectors and IBD), and environmental (dissolved oxygen and salinity) factors within our study system. Although theoretical expectations propose that the homogenizing effects of high gene flow might counteract signatures of local adaptation (Slatkin 1987; Lenormand 2002; Kawecki and Ebert 2004), analogous fine‐scale genomic structuring has been observed in other highly connected non‐model populations within environmentally heterogeneous marine environments (Sandoval‐Castillo et al. 2018; Barceló et al. 2022; Boulanger et al. 2022), including the South African coastline (Nielsen et al. 2020; Klein et al. 2024). In such instances, local adaptation has not only persisted despite high gene flow but has been shown to promote local adaptation by introducing genetic variation and facilitating the spread of advantageous alleles (Garant, Forde, and Hendry 2006; Tigano and Friesen 2016). Specifically, the underlying genomic architecture has been shown to facilitate local adaptation through the selection of tightly linked alleles (Yeaman and Whitlock 2011; Guo et al. 2015; Tigano and Friesen 2016; Shi et al. 2021) induced by chromosomal rearrangements and inversions, as evidenced in both Atlantic cod (Barth et al. 2017) and herring populations (Lamichhaney et al. 2012). Consequently, clusters of adaptive alleles could be shielded from the disrupting effects of recombination induced by a high gene flow environment (Thompson and Jiggins 2014), and potentially inherited together, thereby sustaining existing levels of adaptive variation (Tigano and Friesen 2016). These mechanisms could therefore explain the observed pattern of adaptive differentiation despite the high coastal connectivity observed among *S. zygaena* sample populations.

### 4.3 Environmental Drivers of Adaptive Differentiation

Estimates from the present analysis suggest that adaptive variation could, in part, be attributed to environmental factors. Specifically, Genotype‐Environment Association (GEA) analysis revealed significant correlations between genetic variation and environmental gradients, particularly dissolved oxygen and salinity. While such associations with a salinity gradient have been previously proposed in other marine species (Ubeda, Simpfendorfer, and Heupel 2009; Froeschke, Stunz, and Wildhaber 2010; Ward‐Paige et al. 2014) including the blue skate *Dipturus batis* (Delaval et al. 2022), the copper shark *C. brachyurus* (Klein et al. 2024), and several dolphin species (Barceló et al. 2022; Pratt et al. 2022; Wittwer et al. 2023), limited information exists regarding the relationships between environmental variables and the behavior of *S. zygaena* . However, most marine elasmobranchs, including *S. zygaena* , generally exhibit a narrow tolerance for salinity fluctuations due to the high energetic costs associated with maintaining a hyperosmotic state relative to the surrounding seawater (Smith 1931). To prevent excessive salt accumulation, these species rely on specialized structures, such as the rectal glands, to actively expel excess salts and maintain osmotic balance (Piermarini and Evans 2000). In addition to managing potential physiological stressors (Heupel and Simpfendorfer 2008; Dowd et al. 2010; Tunnah et al. 2016), movement within specific salinity ranges could also serve as a strategy for predator avoidance or maximizing prey availability (Heupel and Hueter 2002; Ubeda, Simpfendorfer, and Heupel 2009; Pillans et al. 2020).

Across the regional species distribution of *S. zygaena* , the Agulhas Current has been shown to significantly shape oceanographic conditions along the south‐east coast of South Africa (Lutjeharms et al. 2001; Popova et al. 2016; Rouault, Pohl, and Penven 2010), with prominent upwelling observed at the northern end of the Natal Bight (Lutjeharms, Valentine, and Van Ballegooyen 2000) and across the Agulhas Bank (Lutjeharms et al. 1996; Goschen et al. 2015). These upwelling processes transport nutrient‐rich water to the surface, significantly affecting productivity and nutrient concentrations in the region (Lutjeharms et al. 1996; Lutjeharms, Cooper, and Roberts 2000; Barlow et al. 2010). Moreover, seasonal rainfall and subsequent increased river outflow have been evidenced to affect regional salinity concentrations (Russo et al. 2019). As a result, localized fluctuations in dissolved oxygen and salinity could occur (Figure S4) (Meyer, Lutjeharms, and de Villiers 2002; Russo et al. 2019), potentially impacting the distribution and abundance of marine species, including *S. zygaena* and its associated prey species (Roberts 2005; Ubeda, Simpfendorfer, and Heupel 2009; Drymon et al. 2013; Jacobs et al. 2022). Furthermore, such fluctuations are particularly pronounced in nearshore environments, which are critical nursery habitats for many species, including juvenile *S. zygaena* (Smale 1991; Afonso et al. 2022; Albano et al. 2023). Consequently, these environmental influences could, in turn, account for the observed clinal pattern of adaptive differentiation identified in the present study, although future analyses are required to conclusively establish this.

Interestingly, the genetic cline observed in the present study also correlated with a temperature gradient (Figure S4), consistent with some studies suggesting thermal heterogeneity as a potential determinant of habitat selection and distribution of *S. zygaena* populations (Diemer, Mann, and Hussey 2011; Dicken et al. 2018; Santos and Coelho 2018; Logan et al. 2020). The south to east coastline can broadly be characterized into several biogeographical zones: the subtropical east and the warm to cold temperate south (Figure 1), each defined by a unique temperature profile (Bustamante and Branch 1996; Turpie, Beckley, and Katua 2000; Potts, Götz, and James 2015). The degree of genetic differentiation observed among populations within these bioregions, namely the subtropical east (JB, AB, KZS, KZC, and KZN) and the warm to cold temperate south (FB, STR, WT, and MB), could be attributed to temperature variations between these respective bioregions (Figure 5). Consequently, even though the RDA analysis failed to detect a significant association with temperature, its potential influence cannot entirely be disregarded (Capblancq and Forester 2021). Moreover, it is important to recognize that marine systems are inherently complex (Barry and Dayton 1991; Hood, Beckley, and Wiggert 2017), and the spatial and temporal variability of various environmental covariates in addition to salinity and dissolved oxygen, could contribute to the observed patterns of adaptive variation. The present analysis focused exclusively on sea‐surface environmental variables (Table S2), and therefore the dynamics of the marine environment may not have been fully captured, potentially contributing to the residual variance in the global RDA model. Alternatively, it is likely that adaptation only influences a small fraction of the genome (Bay et al. 2017), and that adaptive traits are controlled by many loci of small effect (Savolainen, Lascoux, and Merilä 2013; Yeaman 2015), some of which may not have been detected owing to the restriction enzyme‐based method employed in the present analysis (Hoban et al. 2016). Furthermore, while statistically significant, the selected environmental variables only explain a small portion of the overall genomic variation. Therefore, while dissolved oxygen and salinity accounted for some genomic variation, the inclusion of distance vectors (MEM38, MEM47, MEM50, and MEM51), along with the significant IBD assessment, suggests that a notable portion of variance could also be influenced by spatial factors. It is crucial to acknowledge, however, that a more balanced sampling design, especially incorporating a continuous sampling scheme (e.g., incorporating samples from the region between AB and KZS), would significantly enhance future research to decisively differentiate the effects of spatial and environmental variables on genomic variation. Alternatively, considering the intricate interplay between spatial and environmental factors that may account for the observed levels of differentiation, it is plausible that such interactions may not be adequately captured by a linear model such as RDA (Capblancq and Forester 2021).

## 5 Conclusion

This study employed 3RAD whole‐genome SNP data to investigate the regional population genomics of the globally distributed smooth hammerhead shark, *S. zygaena* . This investigation suggests that populations from the south to east coast of South Africa are likely highly connected, influenced by high gene flow and minimal dispersal barriers. A pronounced genetic cline from south to east was evident based on putatively adaptive SNPs, signifying the presence of differentiation selection pressures within a limited spatial range. Recognizing south to east *S. zygaena* populations as a single genetic stock is crucial, as is considering their unique adaptations to different habitats along South Africa's south to east coast. Furthermore, conservation efforts should extend beyond the current study area to explore adaptations across other regions of *S. zygaena* 's distribution range, focusing on the identification of specific adaptations that enable their persistence in diverse marine environments. These investigations are critical for guiding future molecular research aimed at understanding the molecular basis of adaptation and identifying the best practices for safeguarding the evolutionary potential of the vulnerable hammerhead shark species, *S. zygaena* . The present study contributes to the growing body of evidence exploring the genetic basis of local adaptation in hammerhead sharks and provides a foundation for future research into the intricate interplay between genetic diversity, adaptation, and the rapidly changing marine environment.

## Author Contributions

**D. L. Grobler:** conceptualization (supporting), data curation (lead), formal analysis (lead), funding acquisition (supporting), methodology (supporting), project administration (supporting), resources (supporting), software (supporting), validation (lead), visualization (lead), writing – original draft (lead), writing – review and editing (lead). **J. D. Klein:** conceptualization (lead), data curation (supporting), formal analysis (supporting), funding acquisition (lead), investigation (supporting), methodology (supporting), project administration (lead), resources (lead), supervision (equal), writing – original draft (supporting), writing – review and editing (supporting). **M. L. Dicken:** resources (lead), writing – review and editing (supporting). **K. Mmonwa:** writing – review and editing (supporting). **M. Soekoe:** resources (lead), writing – review and editing (supporting). **M. van Staden:** resources (lead), writing – review and editing (supporting). **S. B. Hagen:** data curation (supporting), writing – review and editing (supporting). **S. N. Maduna:** data curation (supporting), formal analysis (supporting), methodology (supporting), resources (supporting), writing – review and editing (supporting). **A. E. Bester‐van der Merwe:** conceptualization (lead), data curation (supporting), formal analysis (supporting), funding acquisition (lead), investigation (lead), methodology (supporting), project administration (lead), resources (supporting), supervision (lead), writing – original draft (supporting), writing – review and editing (supporting).

## Conflicts of Interest

The authors declare no conflicts of interest.

## Supporting information


**Figure S1.** Venn diagram illustrating the number of putatively adaptive loci identified using OUTFlank, pcadapt and Redundancy Analysis (RDA).
**Figure S2.** Cross Entropy Criterion plot generated in LEA for determining the optimal number of ancestral *Sphyrna zygaena* populations for (A) selectively neutral (*n* = 111, 243), and (B) putatively adaptive loci (*n* = 4844).
**Figure S3.** The optimal number of genetic clusters inferred for *Sphyrna zygaena* samples, ranked according to the Bayesian Information Criterion (BIC) for (A) selectively neutral (*n* = 111, 243), and (B) putatively adaptive loci (*n* = 4844), respectively.
**Figure S4.** Fluctuations in (A) Mean sea‐surface temperature (MS_biogeo13_sst_mean_5m), (B) maximum dissolved oxygen concentration at the sea surface (BO22_dissoxmax_ss), and (C) mean sea‐surface salinity (MS_biogeo08_sss_mean_5m) of southern Africa. Bioclimatic data was obtained from either the Marine Spatial Ecology (MARSPEC; MS) or Bio‐ORACLE (BO) database.

**Table S1.** Collection data from 95 *Sphyrna zygaena* samples collected along the south to east coast of South Africa. To note, all sampled *S. zygaena* represent juveniles based on precaudal length (cm) (Gallagher and Klimley, 2018).
**Table S2.** Environmental variables obtained from Bio‐ORACLE and Marine Spatial Ecology (MARSPEC) databases.
**Table S3.** Collinearity estimates (Variance Inflation Factor, VIF) of the final partial RDA model considering the full SNP dataset for all sampled *Sphyrna zygaena* populations, as determined by vegan.
**Table S4.** Summary of the number of genetic clusters (*K*) present within sampled *Sphyrna zygaena* (*n* = 93) along the south to east coast of South Africa.

## Data Availability

Raw sequence files can be accessed via dryad (Private for Peer Review). All code and scripts utilized toward the completion of the present study can be viewed on Github (https://github.com/dylangrblr; Private for Peer Review).
