# Supplementary material for: Seascape Genomics of the Smooth Hammerhead Shark Sphyrna zygaena Reveals Regional Adaptive Clinal Variation
Source: Ecol Evol. 2024 Dec 12;14(12):e70644. doi: 10.1002/ece3.70644 (PMC11635309; doi:10.1002/ece3.70644)
Supplement: Supplementary file 1 — Figure S1. Venn diagram illustrating the number of putatively adaptive loci identified using OUTFlank, pcadapt and Redundancy Analysis (RDA). Figure S2. Cross Entropy Criterion plot generated in LEA for determining the optimal number of ancestral Sphyrna zygaena populations for (A) selectively neutral (n = 111, 243), and (B) putatively adaptive loci (n = 4844). Figure S3. The optimal number of genetic clusters inferred for Sphyrna zygaena samples, ranked according to the Bayesian Information Criterion (BIC) for (A) selectively neutral (n = 111, 243), and (B) putatively adaptive loci (n = 4844), respectively. Figure S4. Fluctuations in (A) Mean sea‐surface temperature (MS_biogeo13_sst_mean_5m), (B) maximum dissolved oxygen concentration at the sea surface (BO22_dissoxmax_ss), and (C) mean sea‐surface salinity (MS_biogeo08_sss_mean_5m) of southern Africa. Bioclimatic data was obtained from either the Marine Spatial Ecology (MARSPEC; MS) or Bio‐ORACLE (BO) database. [file ECE3-14-e70644-s002.pdf]

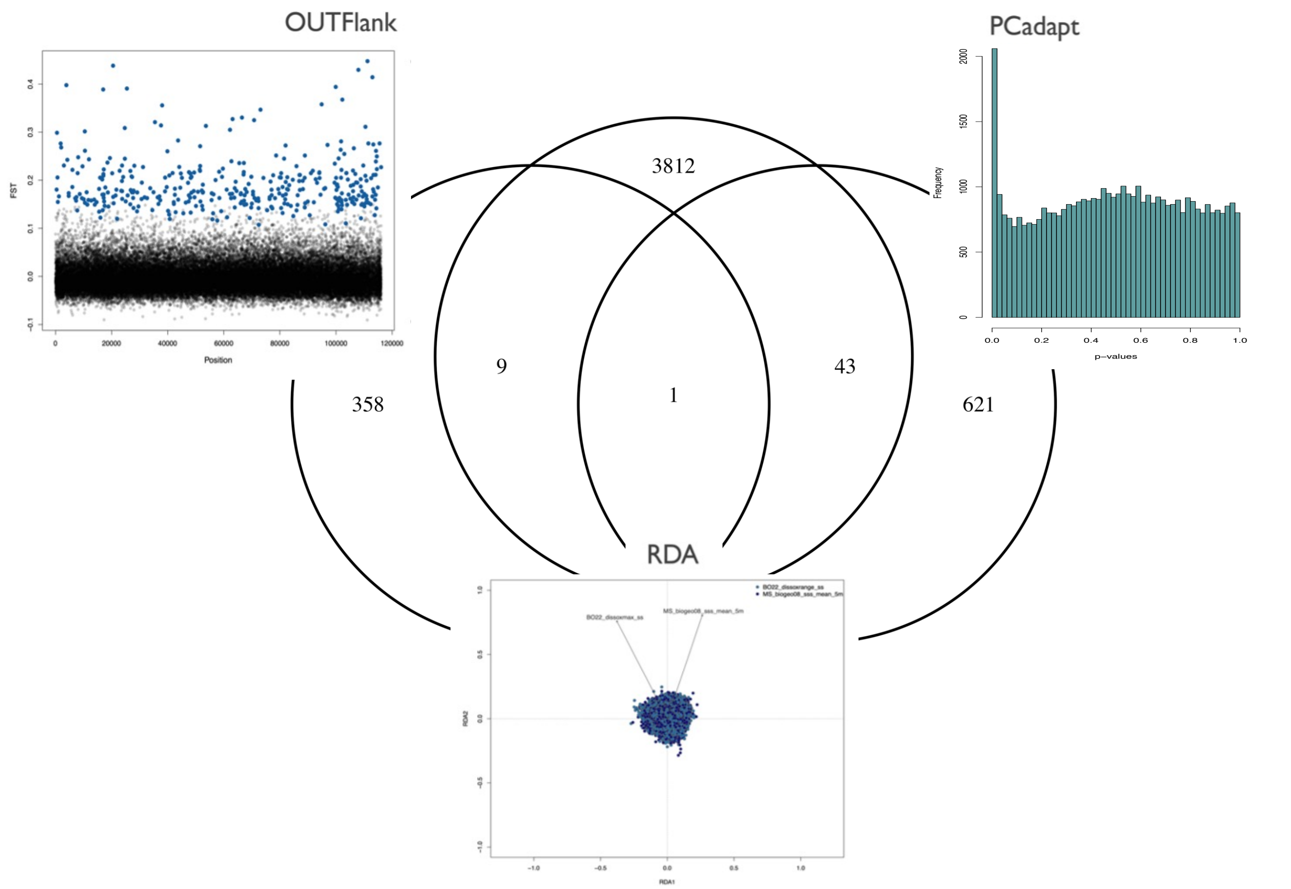
OUTFlank
PCadapt
RDA
3812
9
43
1
358
621
FST
Position
Frequency
p−values
RDA1
RDA2
BO22_dissoxrange_ss
MS_biogeo08_sss_mean_5m
BO22_dissoxmax_ss

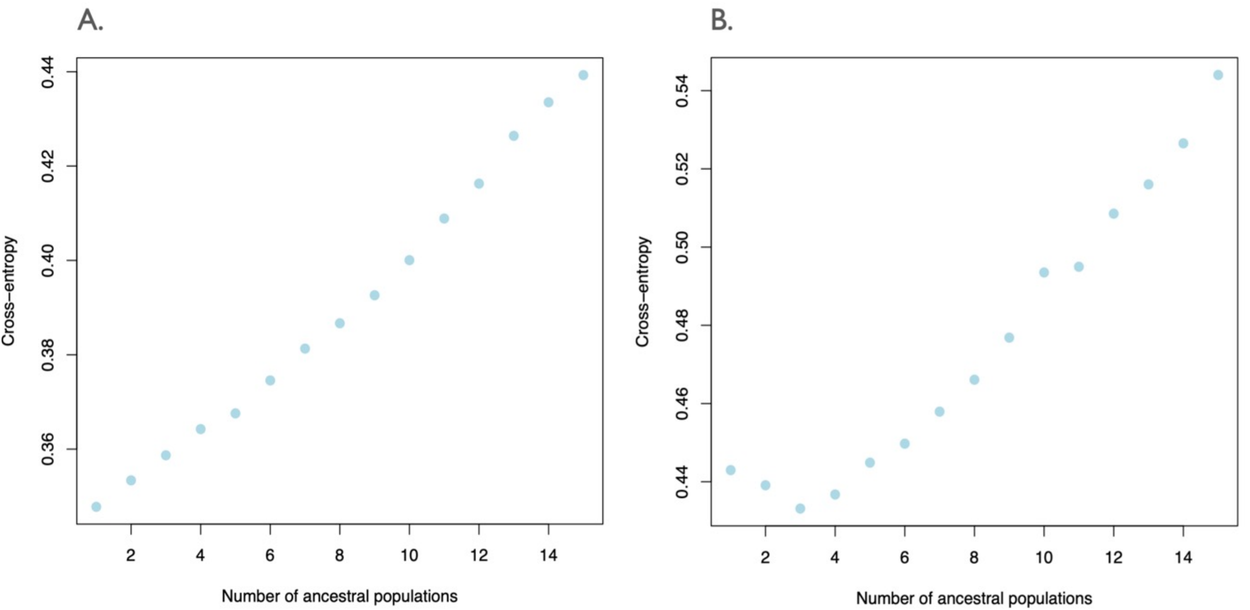
A.
Cross-entropy
0.36
0.38
0.40
0.42
0.44
2
4
6
8
10
12
14
Number of ancestral populations
B.
Cross-entropy
0.44
0.46
0.48
0.50
0.52
0.54
2
4
6
8
10
12
14
Number of ancestral populations

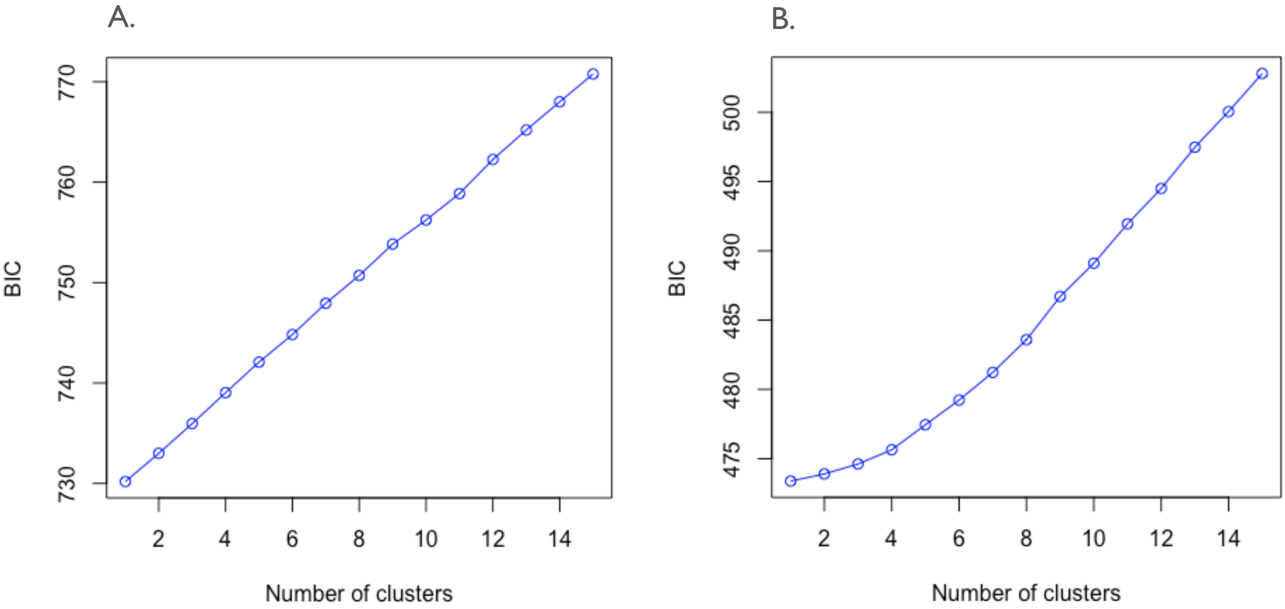
A.
B.
BIC
Number of clusters
730
740
750
760
770
475
480
485
490
495
500
2
4
6
8
10
12
14

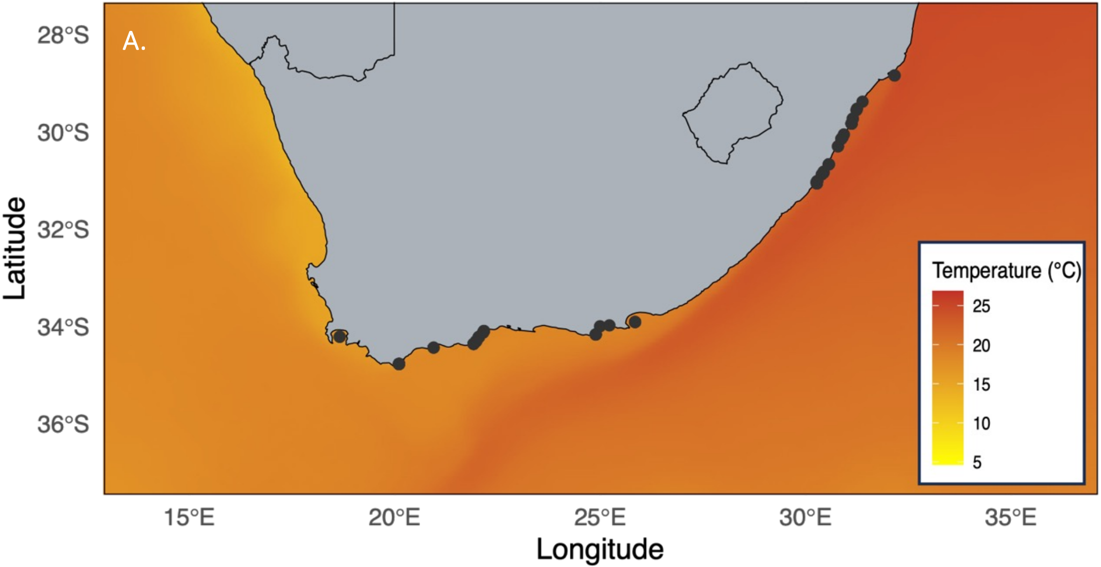
A.
28°S
30°S
32°S
34°S
36°S
Latitude
15°E
20°E
25°E
30°E
35°E
Longitude
Temperature (°C)
25
20
15
10
5

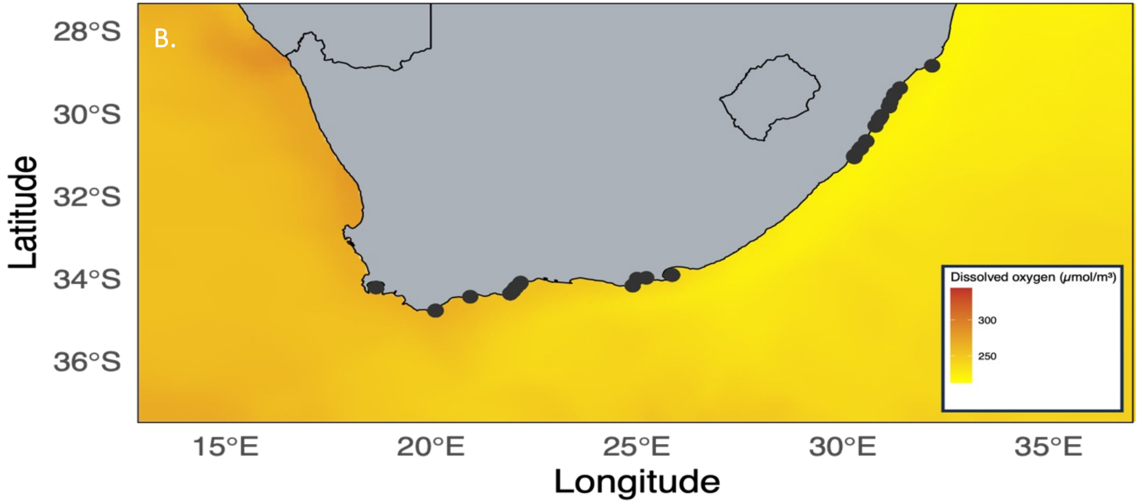
B.
28°S
30°S
32°S
34°S
36°S
Latitude
15°E
20°E
25°E
30°E
35°E
Longitude
Dissolved oxygen (μmol/m³)
300
250

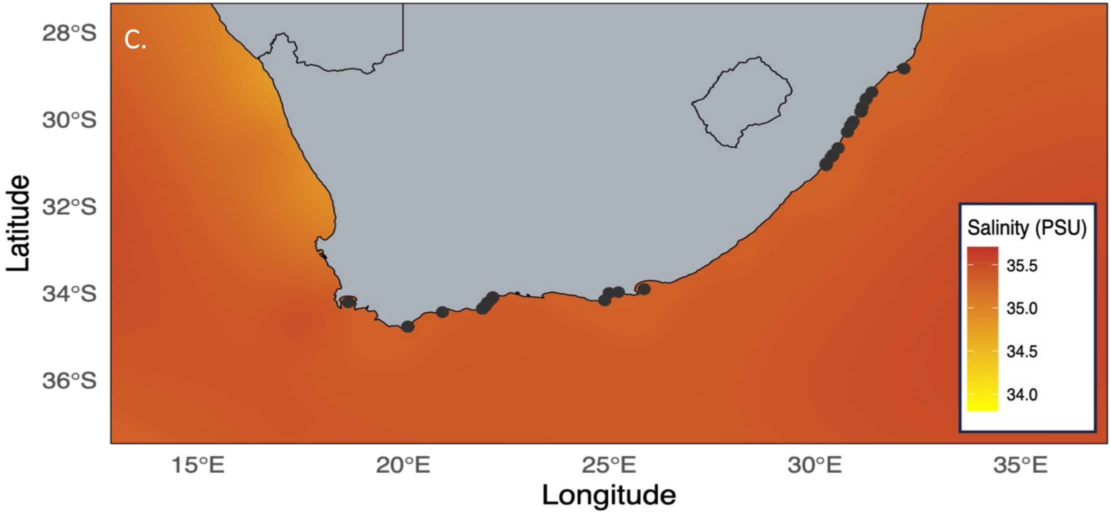
C.
28°S
30°S
32°S
34°S
36°S
Latitude
15°E
20°E
25°E
30°E
35°E
Longitude
Salinity (PSU)
35.5
35.0
34.5
34.0
