# Supplementary material for: Seascape Genomics of the Smooth Hammerhead Shark Sphyrna zygaena Reveals Regional Adaptive Clinal Variation
Source: Ecol Evol. 2024 Dec 12;14(12):e70644. doi: 10.1002/ece3.70644 (PMC11635309; doi:10.1002/ece3.70644)
Supplement: Supplementary file 2 — Table S1. Collection data from 95 Sphyrna zygaena samples collected along the south to east coast of South Africa. To note, all sampled S. zygaena represent juveniles based on precaudal length (cm) (Gallagher and Klimley, 2018). Table S2. Environmental variables obtained from Bio‐ORACLE and Marine Spatial Ecology (MARSPEC) databases. Table S3. Collinearity estimates (Variance Inflation Factor, VIF) of the final partial RDA model considering the full SNP dataset for all sampled Sphyrna zygaena populations, as determined by vegan. Table S4. Summary of the number of genetic clusters (K) present within sampled Sphyrna zygaena (n = 93) along the south to east coast of South Africa. [file ECE3-14-e70644-s001.docx]

15. Supplementary material

Insert Figure_S1 – Figure_S4 here

**Table S1.** Collection data from 95 *Sphyrna zygaena* samples collected along the south to east coast of South Africa. To note, all sampled *S. zygaena* represent juveniles based on precaudal length (cm) (Gallagher and Klimley, 2018). Missing entries are indicated by “-“ and sex, as “M” and “F” for male and female, respectively. Location labels denote: False Bay (FB), Struisbaai (STR), Witsand (WT), Mossel Bay (MB), Jeffreys Bay (JB), Algoa Bay (AB), KwaZulu-Natal South (KZS), KwaZulu-Natal Central (KZC), and KwaZulu-Natal North (KZN).

| Sample ID | Area | Location | Size (PCL in cm) | Sex | Provided by | Collection date |
| --- | --- | --- | --- | --- | --- | --- |
| CM1 | Gordan’s Bay | FB | 101 | M | Reel Science Co. | 31/12/2020 |
| JM1 | Gordan’s Bay | FB | 102 | F | Reel Science Co. | 20/03/2021 |
| MS21 | Die Plaat | STR | 83 | F | Reel Science Co. | 27/03/2020 |
| MS23 | Die Plaat | STR | 93 | F | Reel Science Co. | 27/03/2020 |
| MS25 | Die Plaat | STR | 73 | F | Reel Science Co. | 27/03/2020 |
| MS26 | Die Plaat | STR | 87 | F | Reel Science Co. | 27/03/2020 |
| MS27 | Die Plaat | STR | 82 | F | Reel Science Co. | 27/03/2020 |
| MS28 | Die Plaat | STR | 92 | F | Reel Science Co. | 27/03/2020 |
| JB1 | Die Plaat | STR | 74 | F | Reel Science Co. | 19/12/2020 |
| JB2 | Die Plaat | STR | 64 | M | Reel Science Co. | 19/12/2020 |
| JB3 | Die Plaat | STR | 70 | F | Reel Science Co. | 09/01/2020 |
| BC1 | Die Plaat | STR | 84 | F | Reel Science Co. | 23/01/2021 |
| BC2 | Die Plaat | STR | 88 | F | Reel Science Co. | 23/01/2021 |
| RP1 | Die Plaat | STR | 91 | F | Reel Science Co. | 23/01/2021 |
| CM2 | Die Plaat | STR | 90 | F | Reel Science Co. | 13/02/2021 |
| CM3 | Die Plaat | STR | 109 | F | Reel Science Co. | 13/02/2021 |
| GB1 | Die Plaat | STR | 97 | F | Reel Science Co. | 13/02/2021 |
| GB2 | Die Plaat | STR | 99 | F | Reel Science Co. | 13/02/2021 |
| GB3 | Die Plaat | STR | 76 | M | Reel Science Co. | 13/02/2021 |
| GB4 | Die Plaat | STR | 75 | M | Reel Science Co. | 13/02/2021 |
| GB5 | Die Plaat | STR | 88 | F | Reel Science Co. | 13/02/2021 |
| JM2 | Die Plaat | STR | 102 | F | Reel Science Co. | 20/03/2021 |
| BC3 | Infanta | WT | 93 | M | Reel Science Co. | 06/02/2021 |
| BC4 | Infanta | WT | 83 | F | Reel Science Co. | 06/02/2021 |
| SZ2 | Hartenbos | MB | 74 | - | Michaela van Staden | 12/2019 |
| SZ3 | Dana Bay | MB | 102 | - | Michaela van Staden | 12/2019 |
| SZ4 | Dana Bay | MB | 74 | - | Michaela van Staden | 12/2019 |
| SZ5 | Dana Bay | MB | 112 | - | Michaela van Staden | 12/2019 |
| SZ6 | Dana Bay | MB | 46 | - | Michaela van Staden | 12/2019 |
| SZ7 | - | MB | 114 | - | Michaela van Staden | 12/2019 |
| SZ8 | Saal | MB | 108 | F | Michaela van Staden | 12/12/2019 |
| SZ9 | Saal | MB | 129 | F | Michaela van Staden | 12/12/2019 |
| SZ10 | Saal | MB | 66 | F | Michaela van Staden | 13/12/2019 |
| SZ12 | Klein Brak Rivier | MB | 66 | - | Michaela van Staden | 22/12/2019 |
| SZ13 | Gouritz | MB | 90 | - | Michaela van Staden | 21/12/2019 |
| SZ14 | Fransmanshoek | MB | 94 | - | Michaela van Staden | 20/12/2019 |
| SZ15 | Gouritz | MB | 67 | - | Michaela van Staden | 22/12/2019 |
| SZ16 | Fransmanshoek | MB | - | F | Michaela van Staden | 19/12/2019 |
| SZ17 | Fransmanshoek | MB | - | F | Michaela van Staden | 19/12/2019 |
| SZ18 | Gouritz | MB | 77 | - | Michaela van Staden | 22/12/2019 |
| SZ19 | Gouritz | MB | 61 | - | Michaela van Staden | 21/12/2019 |
| SZ20 | Gouritz | MB | 81 | - | Michaela van Staden | 20/12/2019 |
| SZ21 | Gouritz | MB | 79 | - | Michaela van Staden | 20/12/2019 |
| SZ22 | Klein Brak Rivier | MB | 72 | F | Michaela van Staden | 26/02/2020 |
| SZ23 | Fransmanshoek | MB | 100 | - | Michaela van Staden | 02/2020 |
| SZ25 | Klein Brak Rivier | MB | 72 | F | Michaela van Staden | 26/02/2020 |
| DVB6 | van Stadens | JB | 103 | F | South African Shark Conservancy | 2013 |
| JB227 | Kabeljouw | JB | 108 | - | Reel Science Co. | 18/12/2020 |
| DVB16 | St. Francis | JB | - | F | Reel Science Co. | 23/12/2020 |
| DVB17 | St. Francis | JB | 97 | F | Reel Science Co. | 23/12/2020 |
| DVB19 | St. Francis | JB | 86 | F | Reel Science Co. | 23/01/2021 |
| DVB20 | St. Francis | JB | 107 | F | Reel Science Co. | 13/01/2021 |
| DVB21 | St. Francis | JB | 96 | F | Reel Science Co. | 13/01/2021 |
| SZ1 | Kabeljouw | JB | 78 | F | Michaela van Staden | 20/03/2019 |
| AB2 | Algoa Bay | AB | - | - | Matt Dicken | 03/07/2015 |
| AB5 | Algoa Bay | AB | - | - | Matt Dicken | 03/07/2015 |
| AB6 | Algoa Bay | AB | - | - | Matt Dicken | 03/07/2015 |
| AB8 | Algoa Bay | AB | - | - | Matt Dicken | 11/02/2014 |
| AB10 | Algoa Bay | AB | - | - | Matt Dicken | 11/08/2014 |
| AMA18003 | eManzimoti | KZS | 95 | M | KwaZulu-Natal Sharks Board | 04/12/2018 |
| WIN18006 | Winklespruit | KZS | 88 | M | KwaZulu-Natal Sharks Board | 27/11/2018 |
| UMG15018 | Umgababa | KZS | 96 | M | KwaZulu-Natal Sharks Board | 29/12/2015 |
| UMG16008 | Umgababa | KZS | 89 | M | KwaZulu-Natal Sharks Board | 22/06/2016 |
| SCO16002 | Scottburgh | KZS | 93 | M | KwaZulu-Natal Sharks Board | 08/01/2016 |
| SCO18013 | Scottburgh | KZS | 87 | F | KwaZulu-Natal Sharks Board | 05/12/2016 |
| BAN16005 | Banana Beach | KZS | 99 | F | KwaZulu-Natal Sharks Board | 20/06/2016 |
| BAN16006 | Banana Beach | KZS | 95 | M | KwaZulu-Natal Sharks Board | 16/09/2016 |
| BAN16011 | Banana Beach | KZS | 100 | M | KwaZulu-Natal Sharkss Board | 02/11/2016 |
| BAN16013 | Banana Beach | KZS | 93 | M | KwaZulu-Natal Shark Board | 26/01/2016 |
| ST16001 | Banana Beach | KZS | 94 | F | KwaZulu-Natal Sharks Board | 04/07/2016 |
| UVO16003 | Uvongo | KZS | 88 | M | KwaZulu-Natal Sharks Board | 24/05/2021 |
| UVO20004 | Uvongo | KZS | 100 | F | KwaZulu-Natal Sharks Board | 24/12/2020 |
| RAM19001 | Ramsgate | KZS | 88 | M | KwaZulu-Natal Sharks Board | 06/03/2019 |
| GLN16004 | Glenmore | KZS | 100 | F | KwaZulu-Natal Sharks Board | 13/04/2016 |
| GLN16019 | Glenmore | KZS | 100 | F | KwaZulu-Natal Sharks Board | 08/09/2016 |
| T.O16003 | T.O Strand | KZS | 100 | M | KwaZulu-Natal Sharks Board | 15/03/2016 |
| PE15003 | Port Edward | KZS | 88 | M | KwaZulu-Natal Sharks Board | 20/07/2015 |
| PE15004 | Port Edward | KZS | 90 | F | KwaZulu-Natal Sharks Board | 30/09/2015 |
| PE16003 | Port Edward | KZS | 98 | F | KwaZulu-Natal Sharks Board | 26/04/2016 |
| PE19003 | Port Edward | KZS | 75 | F | KwaZulu-Natal Sharks Board | 19/10/2019 |
| DUR15024 | Durban | KZC | 88 | M | KwaZulu-Natal Sharks Board | 04/08/2015 |
| DUR16021 | Durban | KZC | 96 | M | KwaZulu-Natal Sharks Board | 12/07/2016 |
| DUR17014 | Durban | KZC | 96 | F | KwaZulu-Natal Sharks Board | 08/08/2017 |
| DUR17018 | Durban | KZC | 100 | F | KwaZulu-Natal Sharks Board | 06/09/2017 |
| DUR18037 | Durban | KZC | 86 | M | KwaZulu-Natal Sharks Board | 26/11/2018 |
| DUR18038 | Durban | KZC | 85 | M | KwaZulu-Natal Sharks Board | 26/11/2018 |
| DUR19008 | Durban | KZC | 95 | M | KwaZulu-Natal Sharks Board | 15/02/2019 |
| DUR19014 | Durban | KZC | 93 | M | KwaZulu-Natal Sharks Board | 05/03/2019 |
| RB08027 | Richard’s Bay | KZN | 100 | F | KwaZulu-Natal Sharks Board | 02/04/2008 |
| RB15030 | Richard’s Bay | KZN | 105 | F | KwaZulu-Natal Sharks Board | 14/09/2014 |
| BLY16015 | Blythedale | KZN | 72 | M | KwaZulu-Natal Sharks Board | 07/06/2016 |
| TON20003 | Thompson’s Bay | KZN | 95 | M | KwaZulu-Natal Sharks Board | 14/12/2020 |
| BAL16008.2 | Balito Bay | KZN | 80 | F | KwaZulu-Natal Sharks Board | 15/04/2016 |
| UMH16003 | Umhlanga | KZN | 93 | F | KwaZulu-Natal Sharks Board | 14/03/2016 |
| UMH16006 | Umhlanga | KZN | 96 | F | KwaZulu-Natal Sharks Board | 24/10/2016 |

**Table S2.** Environmental variables obtained from Bio-ORACLE and Marine Spatial Ecology (MARSPEC) databases. Respective dataset and layer codes are indicated. Mean, maximum, minimum and range sea-surface measurements were included when available, and are denoted as *mean*, *min*, *max*, and *range*, respectively.

| Name | Layer code | Resolution (arc degree) | Dataset code |
| --- | --- | --- | --- |
| Chlorophyll concentration | BO22_chlo*max*_ss | 0.08 | Bio-ORACLE |
|  | BO22_chlo*mean*_ss |  |  |
|  | BO22_chlo*min*_ss |  |  |
|  | BO22_chlo*range*_ss |  |  |
| Dissolved oxygen concentration | BO22_dissox*max*_ss | 0.08 | Bio-ORACLE |
|  | BO22_dissox*mean*_ss |  |  |
|  | BO22_dissox*min*_ss |  |  |
|  | BO22_dissoxrange_ss |  |  |
| Current velocity | BO22_curvel*max*_ss | 0.08 | Bio-ORACLE |
|  | BO22_curvel*mean*_ss |  |  |
|  | BO22_curvel*min*_ss |  |  |
|  | BO22_curvel*range*_ss |  |  |
| Primary production | BO22_pp*max*_ss | 0.08 | Bio-ORACLE |
|  | BO22_pp*mean*_ss |  |  |
|  | BO22_pp*min*_ss |  |  |
|  | BO22_pp*range*_ss |  |  |
| pH | BO22_ph | 0.08 | Bio-ORACLE |
| Sea-surface temperature  (Annual mean) | MS_biogeo13_sst_*mean*_5m | 0.04 | MARSPEC |
|  | MS_biogeo16_sst_*range*_5m |  |  |
| Salinity  (Annual mean) | MS_biogeo08_sss_*mean*_5m | 1.00 | MARSPEC |
|  | MS_biogeo11_sss_*range*_5m |  |  |

**Table S3.** Collinearity estimates (Variance Inflation Factor, VIF) of the final partial RDA model considering the full SNP dataset for all sampled *Sphyrna zygaena* populations, as determined by vegan. Final predictor variables include the environmental variables, BO22_dissoxmax_ss, and MS_biogeo08_sss_mean_5m, and the spatial variables, MEM1, MEM38, MEM47, MEM50, and MEM51. Variable(s) excluded due to high collinearity (VIF ~ 10) are marked with an asterisk.

| Predictor variable | VIF |
| --- | --- |
| BO22_dissoxmax_ss | 8.208 |
| MS_biogeo08_sss_mean_5m | 2.524 |
| MEM1* | 9.982* |
| MEM38 | 1.000 |
| MEM47 | 1.000 |
| MEM50 | 1.000 |
| MEM51 | 1.000 |

**Table S4.** Summary of the number of genetic clusters (*K*) present within sampled *Sphyrna zygaena* (n = 93) along the south to east coast of South Africa. Genetic clustering methods included *find.clusters*, the *sNMF* algorithm utilized by the LEA package, and likelihood estimation implemented in fastSTRUCTURE. Results are based on 111,243 selectively neutral and 4,844 putatively adaptive SNP markers.

| Genetic clustering method | Number of genetic clusters (*K*) | |
| --- | --- | --- |
|  | Selectively neutral loci | Putatively adaptive loci |
| *find.clusters* | 1 | 1 |
| *sNMF* algorithm (LEA) | 1 | 3 |
| fastSTRUCTURE | 1 | 2 |
